# Beyond PAD Inhibition: Emerging Avenues and Natural Products for Targeting Citrullination in Immune Diseases

**DOI:** 10.3390/biomedicines14040850

**Published:** 2026-04-08

**Authors:** Qilei Chen, Yuhang Ma, Yingyi Liu, Xiaojie Wang, Guanhua Huang, Yizhao Yang, Joshua Ka-Shun Ko, Hubiao Chen

**Affiliations:** 1 Teaching and Research Division, School of Chinese Medicine, Hong Kong Baptist University, Hong Kong 999077, China; 25494147@life.hkbu.edu.hk (Y.M.); liuyingyi@hkbu.edu.hk (Y.L.); wangxiaojie@hkbu.edu.hk (X.W.); 23483547@life.hkbu.edu.hk (G.H.); 23481978@life.hkbu.edu.hk (Y.Y.); jksko@hkbu.edu.hk (J.K.-S.K.); 2 Institute for Research and Continuing Education, Hong Kong Baptist University, Shenzhen 518000, China; 3 Centre for Cancer and Inflammation Research, School of Chinese Medicine, Hong Kong Baptist University, Hong Kong 999077, China

**Keywords:** protein citrullination, peptidylarginine deiminases (PAD), anti-citrullinated protein antibodies (ACPAs), NETosis, natural products, drug discovery

## Abstract

Immune-mediated inflammatory diseases, such as rheumatoid arthritis, multiple sclerosis, and systemic lupus erythematosus, impose a severe and growing global health burden, where current therapies are limited by poor specificity and significant side effects. The peptidylarginine deiminase (PAD)/citrullination axis, in which protein citrullination catalyzed by PADs drives autoantigen generation and sustains inflammation, has emerged as a critical therapeutic target. This review outlines a comprehensive strategy for targeting this axis using natural products. We first detail the established role of natural compounds as direct PAD inhibitors, covering their chemical diversity, inhibitory mechanisms, and therapeutic applications in disease models. Subsequently, the discussion extends to their broader, indirect modulatory functions, highlighting how these compounds can suppress pathogenic citrullination by regulating upstream processes like NETosis and inflammatory signaling. Furthermore, the review introduces the innovative substrate-centric intervention strategy, which represents a paradigm shift toward shielding key arginine residues on autoantigens, thereby preventing the formation of immunogenic neoepitopes. The translational challenges and future directions for each of these avenues are outlined, addressing persistent obstacles including achieving isoform selectivity and biomarker validation. By integrating these multifaceted strategies, from direct inhibition and indirect modulation to substrate protection, this work provides a strategic roadmap for advancing the next generation of more precise, effective, and safe anti-citrullination therapies, ultimately moving beyond conventional enzyme inhibition toward targeted immunomodulation in immune-mediated inflammatory diseases.

## 1. Introduction

Immune-mediated inflammatory diseases, including rheumatoid arthritis (RA), multiple sclerosis (MS), and systemic lupus erythematosus (SLE), represent a significant and growing global health burden, severely impacting patients’ quality of life and society [1]. The pathogenesis of these complex diseases involves intricate interactions among multiple factors, including genetic susceptibility, immune dysregulation, environmental exposures, hormonal influences, and infections [2]. Conventional immunomodulatory therapies, while effective for many, often lack disease specificity due to their broad mechanisms of action, resulting in non-specific immunosuppression and side effects such as infections and malignancies [3]. Moreover, challenges such as substantial interindividual variability in therapeutic responses, loss of efficacy with long-term use, and high treatment costs are prevalent. Therefore, there is an urgent need to develop novel therapeutic strategies with improved safety profiles and more precise mechanisms of action [4].

A key molecular event implicated in the pathogenesis of these immune disorders is protein citrullination, a Ca^2+^-dependent post-translational modification (PTM) catalyzed by peptidylarginine deiminases (PADs), which convert positively charged arginine residues into neutral citrulline [5,6]. First reported in 1958 [7], this deamination reaction alters key physicochemical properties: the replacement of arginine (isoelectric point pI ~11.41) with citrulline (pI ~5.91) reduces the protein’s net positive charge, thereby disrupting hydrogen bonding and electrostatic interactions [8,9]. Critically, as citrulline is a non-canonical amino acid not encoded by DNA, its occurrence is exclusively enzymatic. To date, no robust enzymatic decitrullination mechanism has been identified, rendering citrullination irreversible, unlike reversible PTMs such as phosphorylation. This irreversibility facilitates sustained signaling and long-term functional changes [10]. PAD substrates span diverse protein classes, including structural proteins (e.g., keratin, vimentin), cytoskeletal elements (e.g., actin, tubulin), extracellular matrix components (e.g., fibronectin), and histones [11].

Citrullination regulates core cellular processes, including epigenetic gene control, formation of neutrophil extracellular traps (NETs), DNA damage-induced apoptosis, and immune activation. For instance, histone citrullination modulates chromatin structure and transcription [10], while vimentin citrullination can generate neoepitopes that act as autoantigens, influencing immune regulation and evasion. Among these processes, the formation of NETs represents a key mechanism linking PAD activity to immune pathology. However, the requirement of PAD4 in NETosis is stimulus-dependent. While PAD4-mediated histone citrullination is essential for NET formation in response to calcium ionophores and certain microbial stimuli, alternative pathways exist. For instance, PMA-induced NETosis can occur independently of PAD4 activity [12]. Similarly, even in response to opsonized *Candida albicans*, a stimulus that robustly induces histone citrullination, NETosis still occurs in PAD4 knockout mice, indicating that PAD4 is dispensable in this context [13]. Thus, the involvement of PAD4 in NETosis should be interpreted in the context of the specific experimental or pathological setting. Collectively, these mechanisms underscore the critical roles of tightly regulated citrullination in both physiology and pathology, and its dysregulation forms a cornerstone in the pathogenesis of multiple immune diseases. The centrality of the PAD/citrullination axis has made it a compelling therapeutic target; however, the pursuit of effective strategies demands a look beyond direct enzyme inhibition.

In this context, natural products emerge as a uniquely valuable resource for pioneering such novel strategies. Historically, natural products have been a vital source of therapeutic agents, offering unparalleled structural diversity and chemical intricacy compared to synthetic compound libraries [14]. With inherent multi-targeting capabilities, conformational diversity, and unique ability to bind to shallow or complex protein interfaces, natural products possess distinct advantages for modulating intricate biological pathways like those involved in immune dysregulation [15]. Natural products and their derivatives have yielded numerous frontline therapies for inflammatory, infectious, and malignant diseases [16]. This inherent versatility positions natural products ideally for exploring the full therapeutic landscape of the citrullination axis. Their structural and mechanistic diversity supports not only the discovery of direct PAD enzyme inhibitors but also enables the targeting of upstream regulatory pathways. More innovatively, it opens the door to entirely new intervention strategies, such as substrate-directed interventions designed to prevent aberrant citrullination. This multifaceted potential is central to the theme of exploring avenues beyond PAD inhibition.

**Figure 1:**
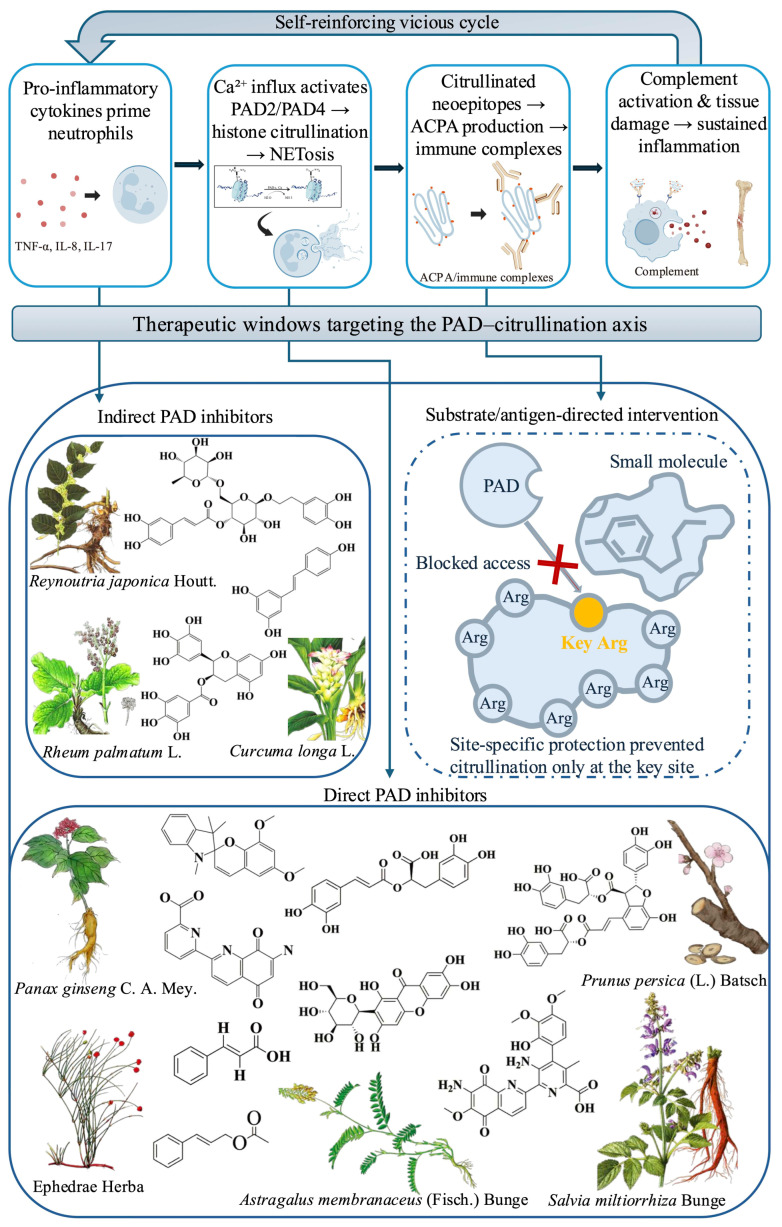
The pathogenic citrullination–inflammation loop and natural product-based intervention strategies. Botanical illustrations and chemical structures presented in this figure are original artwork created for this publication.

Therefore, this review will first delineate the role of citrullination in immune diseases and then critically evaluate natural products as a source for targeting this axis. Our analysis will encompass direct PAD inhibitors, indirect modulators, and innovative, substrate-directed strategies, thereby mapping a comprehensive therapeutic landscape that extends beyond direct enzyme inhibition (Figure 1).

## 2. The Citrullination Pathway: A Therapeutic Target in Immune Diseases

### 2.1. Peptidylarginine Deiminases: The Catalytic Drivers

PADs are a group of five genetically encoded cysteine hydrolases clustered on human chromosome 1p36.1 and mouse chromosome 4E1 [17]. Their catalytic mechanism relies on a conserved active-site cysteine that initiates a nucleophilic attack on the guanidino group of arginine, forming an acyl-enzyme intermediate that hydrolyzes to yield citrulline [6]. Of the five PAD isoforms (PAD1–4, PAD6), PAD6 is catalytically inactive. All human PAD isozymes are ~74–77 kDa proteins composed of 660–665 amino acids, featuring two N-terminal immunoglobulin-like domains and a C-terminal catalytic domain [6,18]. PAD2 and PAD4 can form homodimers with active sites positioned on the same side [19,20]. The catalytic center includes a nucleophilic cysteine (C645 in PAD1 and PAD4, C647 in PAD2, and C646 in PAD3), a histidine (H471), and two aspartate residues (D350 and D473) that stabilize the substrate [21].

PAD activity is highly Ca^2+^-dependent, with 4 to 6 Ca^2+^ binding sites triggering a conformational change that boosts activity by more than 10,000-fold [21,22]. Since activation requires Ca^2+^ concentrations exceeding 100 µM, PADs remain inactive under most physiological resting conditions [6]. Upon Ca^2+^ binding, the active-site cysteine shifts to a catalytically competent position [20,23]. Notably, only PAD2 and PAD4 possess nuclear localization signals, enabling histone citrullination (e.g., H1, H3, H4) [20,24]. Substrate accessibility is further governed by protein secondary structure, explaining why not all arginine residues are susceptible [25].

PAD isoforms exhibit distinct tissue and functional specificity (Table 1). PAD1 and PAD3 are primarily expressed in the epidermis and hair follicles, where they modify keratins and filaggrin; PAD3 additionally targets trichohyalin and vimentin. PAD2, the most widely distributed isoform, is found in the brain, skeletal muscle, and leukocytes, among other tissues, targeting substrates like myelin basic protein (MBP) and histones. PAD4 is predominantly expressed in immune cells (e.g., granulocytes, monocytes, and macrophages) and drives NET formation. PAD6, though inactive, supports female reproductive function [10,26]. Critically, PAD2 and PAD4 are the only isoforms expressed in immune cells, positioning them as key players in immune disease pathogenesis. In addition, the periodontal pathogen *Porphyromonas gingivalis* expresses a unique microbial PAD (PPAD) that catalyzes citrullination in a calcium-independent manner, linking periodontitis to RA through autoantigen generation. PPAD and mammalian PADs are phylogenetically unrelated proteins [27].

### 2.2. The General Core Mechanisms of Citrullination in Immune Diseases

In recent years, protein citrullination has emerged as an important pathological feature of immune diseases, with growing evidence linking to inflammatory responses [5]. Abnormal accumulation of citrullinated proteins is frequently observed across various immune disorders [28]. The shared pathological mechanism typically initiates with a dysregulated increase in intracellular Ca^2+^ within the inflammatory microenvironment, leading to PAD activation and subsequent citrullination of arginine residues on a variety of substrate proteins [29]. Under physiological conditions, immune tolerance to citrullinated proteins is maintained; however, citrullination-induced alterations in protein conformation and folding can expose neoepitopes, breaking immune tolerance and triggering autoimmune responses [30]. Upon release into the extracellular environment, these modified proteins are recognized by antigen-presenting cells and presented as foreign antigens to T cells, initiating a cascade that culminates in anti-citrullinated protein antibody (ACPA) production [31]. ACPAs bind to citrullinated self-antigens, forming immune complexes that exacerbate inflammation and tissue damage [32]. Critically, tissue damage and cell necrosis release intracellular Ca^2+^, sustaining elevated local Ca^2+^ levels and reactivating PADs to produce more citrullinated proteins. This self-reinforcing vicious cycle continuously drives disease progression.

Collectively, this core citrullination-driven pathological cycle manifests across a spectrum of immune diseases, albeit with disease-specific variations in the key PAD isoforms involved, the identity of citrullinated substrates, and the downstream immune responses they provoke. The following sections will detail how these mechanisms operate in specific disease contexts.

### 2.3. Citrullination in Immune Diseases

#### 2.3.1. Rheumatoid Arthritis

RA is a chronic autoimmune disease characterized by symmetric joint inflammation and damage [33]. A hallmark of RA is the abundance of autoantibodies targeting post-translationally modified proteins, with citrullinated antigens playing a central role [28]. In RA joints, PAD2 and PAD4 expression and activity are markedly elevated [34], linked to infiltrating monocytes/macrophages [6,35]. Apoptosis-induced Ca^2+^ release further activates PADs, promoting citrullination of both intra- and extracellular proteins such as vimentin, fibrinogen, and α-enolase and resulting in immune tolerance breakdown [6,28,35]. This triggers the production of ACPAs, which predominantly target a subset of antigens like vimentin and α-enolase. ACPAs promote disease progression by stimulating pro-inflammatory cytokines (e.g., TNF-α), inducing osteoclastogenesis, and activating the complement system [36,37].

Critically, PAD4-driven NET formation citrullinates histones (e.g., H2A, H2B, and H4) and vimentin, releasing antigens that fuel ACPA production. Inflammatory mediators (e.g., TNF-α, IL-8, and IL-17) further promote NETosis, establishing a self-reinforcing vicious cycle [38,39,40]. However, this cycle is not absolute: therapeutic antibodies targeting citrullinated histones H2A/H4 can suppress NET formation, highlighting the potential for targeted interventions [40]. NETs can also directly damage cartilage via PAD2 activity [41]. Furthermore, elevated DKK1 correlates with disease activity and may synergize with PAD-dependent citrullination of type II collagen in driving joint destruction [42].

#### 2.3.2. Multiple Sclerosis

MS is an autoimmune disease characterized by demyelination in the central nervous system (CNS), driven by lymphocyte infiltration and myelin destruction [43]. This ultimately manifests as a range of neurological syndromes and physical disabilities, including sensory and motor deficits, spasticity, fatigue, pain, and cognitive dysfunction [44]. Among key pathogenic mechanisms, hypercitrullination of MBP plays a central role. The 18.5 kDa MBP contains 19 citrullination-susceptible arginine residues [45]; under physiological conditions, ~18% are modified [46], but in MS patients, the ratio rises to ~45% [47]. This hypercitrullination is mediated by PAD2, whose expression is significantly upregulated in MS white matter and peripheral blood [48], associated with hypomethylation of the PAD2 gene promoter [49]. Animal studies confirm PAD2’s necessity: PAD2-knockout mice lack CNS citrullination and are protected from demyelination [50].

Immunologically, citrullinated MBP enhances presentation of the immunodominant peptide MBP 85–99, triggering Th17 responses that exacerbate autoimmune attacks against myelin [51]. Biophysically, excessive citrullination reduces MBP’s net positive charge, causing protein unfolding and impairing myelin compaction [50]. This defect compromises structural integrity and may expose neoepitopes, further fueling autoimmunity and impairing nerve conduction [6,52].

#### 2.3.3. Systemic Lupus Erythematosus

SLE is a chronic autoimmune disease that can cause inflammation and immune-mediated damage across multiple organ systems [53]. A hallmark of SLE is the release of abundant autoantibodies, including ACPAs, which can trigger and sustain the inflammation [54]. Neutrophils are a major source of PAD enzymes in SLE: they express PAD4 on their surface and spontaneously release PAD2 into the extracellular environment, even in the absence of inflammatory stimuli [55,56,57]. These NET-independent processes directly promote extracellular protein citrullination, contributing to elevated citrullinated protein levels in SLE tissues [55]. Additionally, histone H3 can be citrullinated by PADs both intracellularly and extracellularly, further amplifying the pool of immunogenic antigens [55]. PAD4 directly contributes to SLE pathogenesis through its roles in NET formation and histone citrullination, which releases additional autoantigens [58]. High levels of citrullinated proteins further drive immune tolerance loss and massive ACPA production, exacerbating autoimmune responses and highlighting the potential for targeting citrullination pathways in SLE [59].

#### 2.3.4. Psoriasis

Psoriasis is a chronic immune-mediated skin disease characterized by well-demarcated erythematous scaly plaques, pruritus, and pain [5,60]. The expression and activity of PAD1, PAD2, and PAD4 are complexly dysregulated in psoriasis, intricately linked to the immune–inflammatory process [61]. During epidermal differentiation, PAD1 is highly expressed in keratinocyte and citrullinate proteins such as keratin K1 [62], facilitating keratin filament compaction and maintaining epidermal barrier function [47]. Critically, reduced citrullination of epidermal structural proteins like filaggrin and keratins directly impairs epidermal barrier function and promotes inflammation [63]. Although PAD enzymatic activity is enhanced in lesional skin [57], overall protein citrullination is reduced, particularly citrullinated keratin K1 levels, worsening barrier defects and driving disease progression [62].

PAD activity correlates with infiltration of Th17 cells, neutrophils, and dendritic cells, underscoring a close link between citrullination and immune responses [64]. Furthermore, PAD4 drives NET formation by catalyzing histone citrullination and chromatin decondensation, amplifying inflammation in psoriasis [65]. These mechanisms highlight the potential for targeting specific PAD isoforms to restore barrier function or suppress NET-driven pathology.

#### 2.3.5. Inflammatory Bowel Disease

Inflammatory bowel disease (IBD), primarily comprising ulcerative colitis (UC) and Crohn’s disease (CD), is a chronic inflammatory condition characterized by intestinal mucosal inflammation [66]. Citrullination is associated with IBD pathogenesis, with PAD4 being particularly prominent. Clinical analyses reveal elevated PAD4 levels in IBD patients, along with increased citrullination of mitochondrial creatine kinase 1 (CKMT1). This modification reduces CKMT1 stability, triggering its activation via autophagy and ultimately disrupting mitochondrial homeostasis, compromising intestinal barrier integrity and inducing intestinal epithelial cell apoptosis [67]. Notably, IBD incidence is positively correlated with PAD4 expression but negatively correlated with PAD2, suggesting distinct roles for these isoforms [68,69].

Immunohistochemical staining reveals strong PAD2 and PAD4 expression in human UC specimens, and treatment with the PAD inhibitor chloramidine ( Cl-amidine) alleviates inflammation in a UC mouse model, supporting PADs as the therapeutic targets [70]. For clinical differentiation, serum levels of citrullinated vimentin are lower in UC patients compared to CD and non-IBD controls, positioning it as a potential biomarker to distinguish IBD subtypes and improve diagnostic accuracy [71].

#### 2.3.6. Type 1 Diabetes

Type 1 diabetes (T1D) is characterized by insulin-dependent glucose metabolism dysregulation, triggered by islet inflammation and exacerbated by the autoreactive B and T cell responses. Local inflammatory cytokines and reactive oxygen species amplify protein PTMs, including deamidation, oxidation, carbonylation, and citrullination, all of which can breach immune tolerance [72]. In T1D, a series of PTM-modified autoantigens serves as important biomarkers, such as islet cell autoantigen 69 (ICA69), insulin, glutamic acid decarboxylase 65 (GAD65), islet antigen 2 (IA-2), and zinc transporter 8 (ZnT8). Among these, citrullinated glucose-regulated protein 78 (GRP78) and GAD65 trigger robust B and T cell autoimmune responses in both human T1D and the non-obese diabetic (NOD) mouse model, highlighting their pathogenic role [26,73,74]. Transcriptomic and proteomic analyses further indicate that PAD2 is highly expressed in pancreatic islets of patients with T1D at the prediabetic stage, suggesting early involvement in disease initiation [75].

Moreover, inflammation selectively induces glucokinase citrullination in the pancreas, but not in the liver. This modification alters glucokinase enzyme kinetics, while inhibition of PAD2/PAD4 can partially restore the impaired insulin secretion mediated by pro-inflammatory cytokines. Collectively, these results suggest that glucokinase citrullination serves not only as a marker of β-cell dysfunction but also as an autoimmune biomarker in T1D, providing a rationale for therapeutic strategies aimed at restoring β-cell metabolic function through PAD inhibition [76,77].

In summary, the citrullination pathway is an important contributing factor in a variety of immune diseases (Table 2).

### 2.4. A Druggable Axis: Windows of Therapeutic Opportunity

From the upstream activation of PADs triggered by inflammation to the midstream citrullination modification of key arginine sites, and further to the downstream release of autoantigens and the continuous amplification of the adaptive immune response, this core pathway demonstrates potential for intervention at multiple points. Theoretically, intervention strategies can precisely target distinct stages of this pathway.

For instance, upstream approaches may involve modulating Ca^2+^ signaling or redox balance to disrupt PAD activation environments, or developing direct PAD inhibitors (e.g., covalent inhibitors targeting the catalytic cysteine) to reduce citrullinated protein generation at the source. Midstream interventions could focus on directly blocking citrullination events, for example, by designing substrate-competitive inhibitors to occupy key arginine sites or using antibodies to neutralize specific citrullinated autoantigens (e.g., citrullinated vimentin in RA) before they trigger immune responses. Downstream, terminating the pathological cycle might require modulating adaptive immune responses, for example, by targeting ACPA-producing B cells or blocking co-stimulatory signals for T cell activation. This multi-node druggability makes the citrullination pathway a highly attractive therapeutic target network.

Although current therapies predominantly rely on broad anti-inflammatory and immunosuppressive agents, targeting the citrullination pathway itself, particularly PAD enzymes, has emerged as a promising direction for developing disease-specific therapeutics. Taken together, these findings provide a clear theoretical framework and mechanistic basis for developing novel therapeutics, including both synthetic compounds and natural products. While synthetic PAD inhibitors have shown promise, their development is often hampered by challenges such as isoform selectivity and off-target toxicity. In contrast, natural products, with their intrinsic chemical complexity and pleiotropic mechanisms, offer a unique advantage as lead compounds, providing valuable chemical scaffolds and mechanistic insights for the rational design of next-generation synthetic or semi-synthetic therapeutics targeting the citrullination axis. Accordingly, in the following section, we focus on the unique advantages of natural products as modulators of the citrullination axis, aiming to provide insights that may guide future development of natural product-based therapeutics.

## 3. Natural Products: A Treasurable Source for Targeting Protein Citrullination

### 3.1. Direct Inhibitors of PAD Enzymes

Natural products, with their structural diversity, multi-targeting capabilities, multi-pathway modulation, and diverse pharmacological functions, represent a valuable source for developing therapies against immune diseases and immunomodulation. Many serve as direct inhibitors of PAD enzymes, offering significant potential for anti-citrullination therapy.

Consistently, a diverse array of natural products, from single compounds to active ingredients derived from complex formulas, have been identified as direct PAD4 inhibitors with therapeutic potential (Table 3). The table aims to provide a consolidated view of the current evidence for each compound. While key available data on efficacy, binding, and mechanism are presented, it should be noted that for many compounds, detailed pharmacological parameters such as comprehensive toxicology, pharmacokinetics, isoform selectivity, and drug–drug interaction profiles are not yet available in the literature. Their absence in the table reflects this current research gap rather than an omission, highlighting an important avenue for future translational studies.

Formononetin (**1**), an isoflavonoid from many botanical herbs such as *Sophora flavescens*, *Astragalus membranaceus*, and *Trifolium pratense*, alleviates atopic dermatitis inhibiting NET formation via PAD4/MPO suppression, as evidenced by reduced citrullinated histone H3 (CitH3) levels, which in turn leads to normalized skin hyperplasia, reduced infiltration of CD3^+^ T cells, mast cells, and neutrophils, and suppressed inflammatory cytokine expression [78,79]. Echinacoside (**2**), a phenylethanoid glycoside from *Rehmannia glutinosa*, directly binds to PAD4 and inhibits histone citrullination, thereby blocking NET release, alleviating the pro-inflammatory and immunosuppressive tumor microenvironment induced by NETs, suppressing epithelial–mesenchymal transition, and ultimately ameliorating chemotherapy-induced NET-mediated metastasis [80,81]. Pentagalloylglucose (PGG, **3**), a polyphenolic compound from Moutan Cortex, potently inhibits PAD4 with an IC_50_ of 4.50 μM. Drug affinity responsive target stability (DARTS) and molecular dynamics simulations confirm PAD4 as its direct target, and molecular docking reveals multiple strong interactions, positioning PGG as a reversible inhibitor and promising lead compound [82,83]. Pyrroloquinoline quinone (**4**), a dietary micronutrient, inhibits PAD4 with over 95% inhibition at 5 μM and an IC_50_ below 4 μM. Kinetic analysis indicates a mixed-mode inhibition mechanism, while DARTS and molecular docking confirm its direct, high-affinity binding to PAD4, highlighting its potential in autoimmune diseases such as RA and diabetes [84,85,86,87]. Berberine (**5**), the main alkaloid of Coptidis Rhizoma, directly binds all four PAD4 isoforms, reducing PAD4 expression and reversing PAD4-mediated macrophage dysfunction involving IRF5-driven activation, thereby ameliorating its pro-malignant effects in disease progression [88,89,90,91]. Traditional Chinese medicine (TCM) formulas are also rich sources of direct PAD4 inhibitors. Er Miao San (EMS), a classic TCM formula for RA, contains phellodendrine (PHE, **6**) and atractylenolide-I (ATL-I, **7**) as main active components. Studies have shown that EMS reduces inflammation and neutrophil infiltration in collagen-induced arthritis (CIA) mice, inhibits NET formation, and decreases PAD4 and citrullinated histone H3 (CitH3) expression in neutrophils. PHE and ATL-I replicate these effects, and molecular docking confirms their direct binding to PAD4 via stable hydrogen bonds, identifying them as direct PAD4 inhibitors [92,93,94]. Similarly, the “Yiqi Huoxue” formula components ginsenoside Rb1 (Rb1, **8**) and ferulic acid (FA, **9**) target the PAD4 axis. Rb1 directly inhibits PAD4 with an IC_50_ of 23.01 μM, while FA binds high mobility group box 1 (HMGB1). HMGB1 is a key factor driving the cyclic formation of NETs [95,96] and has been confirmed as a potent inducer of RA and has garnered significant attention in the context of immune disorders [97,98], acting upstream of PAD4 by promoting its expression and activation in neutrophils. Their combination synergistically suppresses NET formation and CitH3 by interfering with the HMGB1/PAD4 signaling pathway, thereby alleviating inflammatory injury [99,100,101]. These findings demonstrate that active compounds derived from TCM formulas can act as direct PAD4 inhibitors, suppressing NETosis and inflammation. Marine natural products, including MNPD10752 (**17**), haploscleridamine (**18**), and oroidin (**19**), have also been identified as potent PAD4 inhibitors with favorable pharmacokinetic and toxicity profiles. Among them, haploscleridamine (**18**) exhibits strong binding affinity and inhibitory activity against PAD4 mutants, as confirmed by molecular dynamics simulations and free energy calculations, positioning it as a promising lead for autoimmune disease therapy [102].

**Figure 2:**
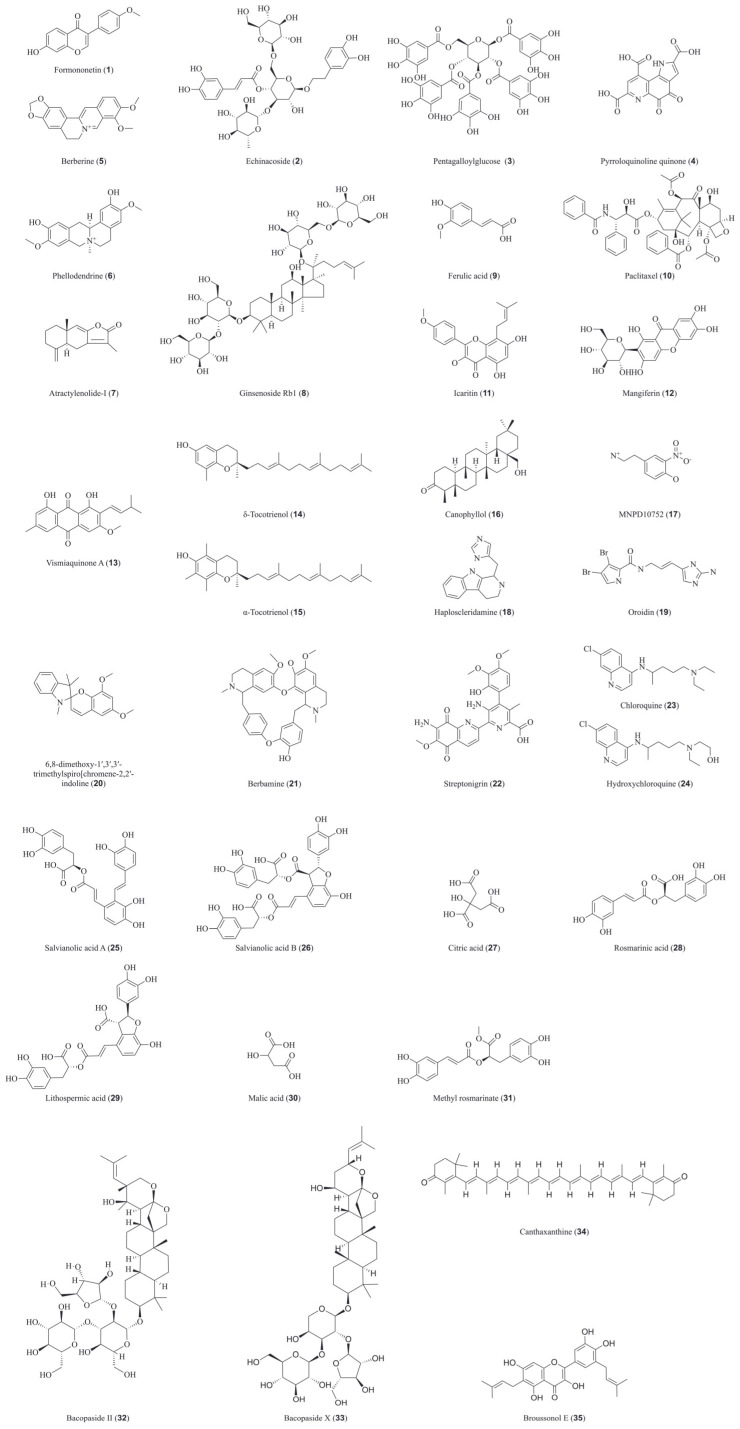
Chemical structures of natural compounds identified as direct inhibitors of the PAD family.

Beyond PAD4, natural products also target other PAD isoforms. Paclitaxel (**10**), a tetracyclic diterpenoid alkaloid isolated from *Taxus*, was first reported in 1998 to inhibit PAD2 via non-competitive binding (*K*_i_: 4.5–10 mM), directly interacting with the monomeric enzyme. It reduces PAD2 activity in demyelinating disease models, and its efficacy is enhanced by vitamin B12, suggesting its therapeutic effects on MS and RA involve blocking arginine citrullination [50,103,104,105,106]. Icaritin (**11**), the active metabolite of icariin from *Epimedium*, directly binds PAD2 at six sites, suppressing suicidal NETosis and PAD2-mediated histone citrullination. This action further disrupts the IL-6/JAK2/STAT3/IL-6 positive feedback loop, limits neutrophil recruitment, and synergizes with anti-PD-1 therapy to counteract T cell exhaustion [107,108]. Other notable examples include inhibitors of bacterial PAD homologs. The methanolic extract of *Cratoxylum cochinchinense* and its active constituents mangiferin (**12**) and vismiaquinone A (**13**) inhibit *Porphyromonas gingivalis* PAD (PPAD), a bacterial PAD homolog linked to periodontitis and RA. Molecular docking confirms their binding to key residues within the PPAD active pocket, supporting their potential as lead compounds for infectious and autoimmune diseases [109,110,111]. Additionally, a spiro[chromene-2,2′-indoline]-based natural product (**20**) and its derivatives inhibit both PAD4 and PAD2 with low micromolar IC_50_ values. A representative derivative significantly ameliorated disease severity in a murine RA model, highlighting the therapeutic potential of this pharmacophore [112]. Collectively, these diverse examples significantly expand the repertoire of PAD-targeting natural products and offer new avenues for drug discovery (Figure 2).

Apart from single compounds, certain herbal extracts also exhibit PAD inhibitory activity (Table 4). Ephedra Herba extract exhibits potent PAD4 inhibition with IC_50_ values of 10–60 μg/mL, while Cinnamomi Ramulus extract achieves 97.7% inhibition at 0.1 mg/mL [87,113]. Extracts from Phellodendri Chinensis Cortex, Salviae Miltiorrhizae Radix Et Rhizoma, Coptidis Rhizoma show weaker activity (IC_50_ > 100 μg/mL) [113]. Despite their moderate potency, these extracts hold research value as natural sources for PAD inhibitor discovery. For instance, berberine (**5**), a direct PAD4 inhibitor (IC_50_: 45.07 μM) that modulates macrophage function, is derived from Coptidis Rhizoma [91]. Extracts from *Rubia cordifolia*, *Isatis indigotica*, and others also exhibit varying degrees of PAD4 inhibition [83]. Collectively, these extracts provide a rich resource for discovering novel PAD inhibitors and advancing therapeutic strategies for autoimmune diseases.

**Table 3:** A comprehensive profile of natural products and derived compounds targeting PADs: Inhibition potency, pharmacological evidence, and mechanistic insights.

Natural Source	Natural Product	Target PAD Isozyme	Validated/Predicted Inhibitor	Biochemical/Cellular Validation Data	Key Pharmacodynamic Outcomes	Proposed Mechanism of Action	Ref.
*Sophora flavescens* (Kushen)	Formononetin (**1**)	PAD4	Validated	- 2,4-Dinitrofluorobenzene-induced atopic dermatitis mouse skin: ↓ PAD4 protein, ↓ citrullinated histone H3-positive (CitH3^+^) & neutrophil extracellular trap (NET) formation [Immunofluorescence (IF), Western blot (WB)]. - Molecular docking (MD): Binding energy = −6.2 kcal/mol.	- 2,4-Dinitrofluorobenzene-induced atopic dermatitis mice (topical, 30 mg/kg): ↑ dermatitis score & skin hyperplasia; ↓ infiltration of CD3^+^ T cells, mast cells, neutrophils; ↓ Il1b, Ccl3, Ccl4; efficacy ~ dexamethasone.	Inhibits PAD4/MPO pathway, suppressing NETosis. Docking suggests direct binding to PAD4.	[79]
*Rehmannia glutinosa*	Echinacoside (**2**)	PAD4	Validated	- Mouse bone marrow neutrophils + ionomycin: ↓ CitH3 & MPO (NETosis) without altering PAD4 expression. - Cellular thermal shift assay (CETSA)/Drug affinity responsive target stability (DARTS): Stabilized PAD4 against thermal/protease degradation). - Microscale thermophoresis: K_D_: 88.1 µM. - MD: Binding energy = −9.1 kcal/mol.	- 4T1 orthotopic breast cancer mouse model (oral, 7.5–30 mg/kg): ↓ Lung metastasis (↓ nodule number/size), neutrophil infiltration & ROS in lungs, NET formation (↓ CitH3, ↓ MPO) in tumors & lungs; Inhibited EMT (↑ E-cadherin, ↓ N-cadherin/vimentin). - Co-culture (4T1 + neutrophils): Reversed NET-promoted cancer cell migration. - Chemotherapy combination model (with cisplatin, oral, 7.5–30 mg/kg): overcame chemotherapy-induced NET accumulation and resistance).	Directly binds PAD4 to inhibit enzymatic activity and NETosis; disrupts NET-driven metastatic niche and EMT.	[81]
Moutan Cortex (Tree Peony bark)	Pentagalloylglucose (**3**)	PAD4	Validated	- Enzymatic inhibition [Color Development Reagent assay (COLDER assay); substrate: N-α-Benzoyl-L-arginine ethyl ester (BAEE)]: IC_50_ = 4.50 μM. - Inhibition kinetics: Mixed-type inhibitor (↓ Km & Vmax). - DARTS: Stabilized PAD4 against protease degradation. - MD: Binding energy = −9.6 kcal/mol. Key interactions with Gln349, His471, Cys645.	/	Potent, reversible, direct inhibitor of PAD4. Acted via mixed-type inhibition kinetics, directly binding to the catalytic site.	[83]
Fruits, vegetables, fermented foods, breast milk	Pyrroloquinoline quinone (**4**)	PAD4	Validated	- Trypsin-assisted immunoassay (substrate: Biotin-R-HisTag): IC_50_ = 3.1 μM. - Enzymatic inhibition (COLDER assay): IC_50_ = 1.6 μM (BAEE), 2.8 μM (L-Arg). - DARTS: Stabilized PAD4 against protease K digestion. - MD: Binding energy = −7.3 kcal/mol.	/	Direct inhibitor of PAD4.	[87]
*Coptis chinensis* (Huanglian) and Coptidis Rhizoma (Chinese Goldthread rhizome)	Berberine (**5**)	PAD4	Validated	- PAD4 citrullination activity ELISA: IC_50_ = 45.07 µM. Citrullination activity was only slightly affected at low doses (2.8–11.2 µM). - DARTS: Stabilized PAD4 against protease K digestion. - MD: Binding energy = −5.48 to −7.45 kcal/mol to multiple PAD4 isomers.	- Urethane & cigarette smoking-induced lung cancer mouse model (oral, 12.5–50 mg/kg): Dose-dependently ↓ lung tumor nodule number; ↓ PAD4 expression and F4/80^+^ macrophage infiltration; reversed M2-like macrophage phenotype (↓CD163/CD206, ↑CD86); improved lung pathology. Efficacy of 25 mg/kg/day ~ GSK484 (4 mg/kg). - Co-culture of PAD4-overexpressed macrophages and A549 cells: Reversed macrophage-promoted lung cancer cell EMT, migration, and anti-apoptosis.	Binds directly to PAD4 but primarily inhibits its protein expression (rather than enzymatic activity) to reverse PAD4-mediated, pro-tumor macrophage polarization (via IRF5 inhibition) and prevent lung carcinogenesis.	[91]
Phellodendri Cortex (Amur Corktree bark)	Phellodendrine (**6**)	PAD4	Validated (in combination with Atractylenolide-I)	- PMA-stimulated mouse bone marrow neutrophils: ↓ CitH3 expression (IF, WB); ↓ PAD4 expression (WB); ↓ MPO-DNA & NE-DNA complexes in supernatant (ELISA). - MD: Binding energy = −6.5 kcal/mol, forms an H-bond with SER-468.	- Collagen-induced arthritis (CIA) mouse model (in combination with Atractylenolide-I, oral, 75:37.5 mg/kg & 150:75 mg/kg): ↓ arthritis index, swollen joint count, paw thickness, serum IL-6 & TNF-α (ELISA), synovial inflammation/hyperplasia/inflammatory cell infiltration (H&E staining), neutrophil infiltration (↓ MPO and NE expression in ankle joints, IHC), bone erosion (micro-CT), blood flow signals in knee joints (Doppler ultrasound). - PMA-stimulated mouse bone marrow neutrophils (in combination with Atractylenolide-I, 100 µM:50 µM): Maintained neutrophil morphology and inhibited NET formation (Scanning Electron Microscopy); Inhibited PMA-induced high expression of MPO and NE (Immunofluorescence).	Inhibits PAD4 (docking suggests direct binding) and reduces its protein expression, thereby decreasing histone citrullination (CitH3) and the formation of neutrophil extracellular traps (NETs), ultimately exerting anti-rheumatoid arthritis effects.	[94]
Atractylodis Macrocephalae Rhizoma (Largehead Atractylodes rhizome)	Atractylenolide-I (**7**)	PAD4	Validated (in combination with Phellodendrine)	- PMA-stimulated mouse bone marrow neutrophils: ↓ CitH3 expression (IF, WB); ↓ PAD4 expression (WB); ↓ MPO-DNA & NE-DNA complexes in supernatant (ELISA). - MD: Binding energy = −7.6 kcal/mol, forms an H-bond with SER-468.			
*Panax ginseng* (Ginseng)	Ginsenoside Rb1 (**8**)	PAD4	Validated	- PAD4 enzymatic inhibition: IC_50_ = 23.01 µM. - PMA-stimulated mouse bone marrow neutrophils: ↓ PAD4 expression (WB). - Activated platelet-stimulated neutrophils: ↓ CitH3^+^ NET formation (IF, ELISA). - MD: binding energy −6.8 kcal/mol.	- Rat myocardial ischemia–reperfusion injury model (oral, 50 mg/kg): ↑ cardiac ejection fractio & fractional shortening; ↓ myocardial infarction area and no-reflow area; ↓ serum CK-MB and cTnI; ↓ microthrombus formation (↓ CD41+ staining); ↓ plasma CitH3. - Ginsnoside Rb1 in combination with ferulic acid (oral, 50 mg/kg each): Synergistically improved myocardial perfusion, reduced NR area, and improved cardiac function compared to either agent alone.	Direct inhibitor of PAD4 enzymatic activity. In combination with FA, inhibits the pathological cascade of platelet HMGB1 release and NET formation, thereby alleviating microvascular obstruction and no-reflow.	[101]
*Ligusticum chuanxiong* Hort. (Chuanxiong)	Ferulic acid (**9**)	PAD4	Validated	- HMGB1 release inhibition (thrombin-stimulated platelets): IC_50_ = 19.28 µM (ELISA). - HMGB1 surface expression inhibition (platelets): IC_50_ = 31.03 µM (Flow cytometry). - PAD4 enzymatic inhibition: IC_50_ = 101.8 µM. - MD: Binding energy = −4.9 kcal/mol (with HMGB1), −6.4 kcal/mol (with PAD4). - WB: Inhibited thrombin-induced p38/ERK1/2 phosphorylation in platelets.		Predominantly inhibits platelet-derived HMGB1 release by suppressing the p38/ERK1/2 pathway, thereby attenuating the pathological cascade of platelet activation, neutrophil-platelet aggregate formation, and subsequent NET formation. Also binds to and inhibits PAD4.	
*Taxus brevifolia*, *T. chinensis*, *T. cuspidata* (Yew tree)	Paclitaxel (**10**)	PAD2	Validated	- Enzymatic inhibition (COLDER assay; BAEE): ~80% inhibition at 12.5 mM - Slot-blot immunoassay (substrate: myelin basic protein): Significant inhibition at 0.5 mM. - Inhibition kinetics: Non-competitive inhibitor. Evidence suggests two substrate-binding sites; paclitaxel inhibits both. - [^3^H]-Paclitaxel binding: Direct binding to PAD monomer (gel filtration).	/	A novel, non-competitive inhibitor of PAD, independent of its microtubule-stabilizing activity. Its inhibitory potency is highly dependent on the substrate (protein vs. small molecule).	[104]
*Epimedii Folium* (Epimedium herb)	Icaritin (**11**)	PAD2	Validated	- Biacore: K_D_ = 61.6 µM - WB: ↓ PADI2 and CitH3 in tumor cells and neutrophils. - MD: Binds PADI2 with high affinity at six potential sites. - RNA-seq & GSVA: Suppressed NET formation and chemokine signaling pathways. - Flow cytometry: ↓ ROS in PMA-stimulated mouse bone marrow neutrophils. - WB: Inhibited MAPK (p38, ERK) and PI3K/AKT pathways in neutrophils.	- Subcutaneous and orthotopic urothelial cancer mouse models (oral, 30 mg/kg): ↓ primary tumor growth, ↓ lung metastasis; ↓ tumor-infiltrating neutrophils (CD11b + Ly6G+); ↓ CitH3 and MPO in tumor tissues. Enhanced CD4^+^ and CD8+ T cell infiltration. - Co-culture (neutrophils + tumor cells): Reversed NET-induced tumor cell invasion, EMT (↓ N-cadherin, ↑ E-cadherin), and stemness (↓ CD44, SOX2, OCT4). - Clinical correlation (human UC samples): High CD66b^+^ neutrophil infiltration correlated with poor prognosis; combination with anti-PD1 showed synergistic tumor suppression in mice.	Direct binder and inhibitor of PAD2. In neutrophils, it suppresses suicidal NETosis by inhibiting PADI2-mediated histone citrullination, ROS generation, and MAPK/PI3K pathways. In tumor cells, it inhibits PADI2, thereby suppressing histone citrullination and transcription of neutrophil-recruiting genes (e.g., IL-6, GM-CSF), disrupting the JAK2/STAT3/IL-6 positive feedback loop.	[108]
*Cratoxylum cochinchinense*	Mangiferin (**12**)	Porphyromonas gingivalis peptidyl arginine deiminase (PPAD)	Predicted	- MD: Binding energy = −3.776 kcal/mol; forms H-bonds with Asp130, Arg154; pi-pi stacking with Trp127.	/	Putative direct binder to the active site of bacterial PPAD.	[111]
	Vismiaquinone A (**13**)	PPAD	Predicted	- MD: Binding energy = −4.071 kcal/mol; forms H-bonds with Asp152, Asp154, and Arg154; pi-pi stacking interaction with Trp127.	/	Ditto	
	δ-Tocotrienol (**14**)	PPAD	Predicted	- MD: Binding energy = −3.122 kcal/mol.	/	Ditto	
	α-Tocotrienol (**15**)	PPAD	Predicted	- MD: Binding energy = −2.723 kcal/mol.	/	Ditto	
	Canophyllol (**16**)	PPAD	Predicted	- MD: Binding energy = −2.900 kcal/mol.	/	Ditto	
Marine natural product	MNPD10752 (**17**)	PAD4	Predicted	- MD: Binding energy = −8.66 kcal/mol.	/	Ditto	[102]
Marine sponge of the Order *Haplosclerida*	Haploscleridamine (**18**)	PAD4	Predicted	- MD: Binding energy = −8.91 kcal/mol.	/	Ditto	
*Agelas oroides*	Oroidin (**19**)	PAD4	Predicted	- MD: Binding energy = −7.26 kcal/mol.	/	Ditto	
Synthetic derivative of chromene-bearing natural products	1′,3′,3′-trimethyl-6,8-dinitrospiro[chromene-2,2′-indoline] (**20**)	PAD4	Validated	- Enzymatic inhibition (fluorometric; BAEE): IC_50_ = 22.5 ± 6.4 μM.	/	Identified as the founding pharmacophore (spiro[chromene-2,2′-indoline] scaffold) for a novel class of PAD4 inhibitors.	[112]
		PAD2	Validated	- Enzymatic inhibition (fluorometric; BAEE): IC_50_ = 26.7 ± 3.8 μM.	/	Demonstrated dual inhibition of PAD4 and PAD2.	
*Berberis vulgaris*	Berbamine (**21**)	PAD4	Validated	- Neutrophils and colon tissue: ↓ PAD4 mRNA and protein expression (qPCR; WB); ↓ CitH3, MPO, and NE expression (IF). - MD: Binding energy = −9.3 kcal/mol; forms H-bonds with Asp632.	- Dextran sulfate sodium (DSS)-induced colitis mouse model (oral, 20 mg/kg): ↓ disease activity index & body weight loss; ↑ colon length; ↓ colonic mucosal ulceration, inflammatory cell infiltration, and crypt damage (H&E); ↓ colonic levels of pro-inflammatory cytokines (TNF-α, IL-1β, IL-6). - Combination study with GSK484, oral, 4 mg/kg): BBM did not provide additional therapeutic benefit, indicating that its effects are dependent on PAD4 inhibition	Binds to and inhibits PAD4 (predicted to be a non-covalent inhibitor), leading to reduced NET formation (↓ CitH3, MPO, NE) in the colon, thereby ameliorating experimental colitis.	[114]
*Streptomyces* species	Streptonigrin (**22**)	PAD4	Validated	- Biochemical substrate assay: IC_50_ = 0.7 ± 0.3 µM. - Selectivity assay: Demonstrated >100-fold selectivity for PAD4 over PAD1, PAD2, and PAD3 (PAD1,2,3 IC_50_ > 100 µM). - Binding mode: Dialysis assay confirmed irreversible inhibition.	/	Irreversible, PAD4-specific inhibitor. Acts as the founding compound for a novel, non-haloacetamidine chemotype of PAD inhibitors, distinct from F-amidine and Cl-amidine.	[115]
Synthetic derivatives of quinine	Chloroquine (**23**)	PAD4	Validated	- Enzymatic inhibition: Dose-dependent inhibition of PAD4 activity. - Specificity: Did not inhibit PAD2, myeloperoxidase, or neutrophil elastase. - Murine neutrophils (PAF or LPS-stimulated): ↓ CitH3 expression. - SPR: KD: 54.1 µM. - MD: Predicted to bind to Arg639 via H-bond.	- Ex vivo NET inhibition (PAF or LPS-stimulated murine bone marrow neutrophils): ↓ cell-free DNA release, indicating reduced NET formation. - Human clinical trial (pancreatic adenocarcinoma patients, chemotherapy + hydroxychloroquine 600 mg twice daily): ↓ serum CitH3 levels compared to chemotherapy alone.	Direct, selective inhibitor of PAD4. Binds to the active site, inhibiting histone citrullination and subsequent NET formation, likely independent of its autophagy inhibitory function.	[116]
	Hydroxychloroquine (**24**)	PAD2	Validated	- Enzymatic inhibition: Dose-dependent inhibition of PAD4 activity. - Specificity: Did not inhibit PAD2, myeloperoxidase, or neutrophil elastase. - Murine neutrophils (PAF or LPS-stimulated): ↓ CitH3 expression. - SPR: *K*_D_: 88.1 µM. - MD: Predicted to bind to Trp347, Ser468, and Glu580 via H-bond.	Ditto	Direct, selective inhibitor of PAD4. Binds to the active site, inhibiting histone citrullination and subsequent NET formation, likely as one of multiple contributing mechanisms (alongside autophagy and TLR9 inhibition).	
Salviae Miltiorrhizae Radix Et Rhizoma (Danshen root)	Salvianolic acid A (**25**)	PAD4	Validated	- Enzymatic inhibition (COLDER assay; BAEE): IC_50_ = 33.52 μM.	/	Potent, reversible, mixed-type inhibitor of PAD4. Predicted to bind via multiple hydrogen bonds.	[117]
	Salvianolic acid B (**26**)	PAD4	Validated	- Enzymatic inhibition (COLDER assay; BAEE): IC_50_ = 721.30 μM.	/	Weak inhibitor of PAD4.	
	Citric acid (**27**)	PAD4	Validated	- Enzymatic inhibition (COLDER assay; BAEE): IC_50_ = 654.60 μM.	/	Weak inhibitor of PAD4.	
	Rosmarinic acid (**28**)	PAD4	Validated	- Enzymatic inhibition (COLDER assay; BAEE): IC_50_ = 321.40 μM.	/	Moderate inhibitor of PAD4.	
	Lithospermic acid (**29**)	PAD4	Validated	- Enzymatic inhibition (COLDER assay; BAEE): IC_50_ = 514.30 μM.	/	Weak inhibitor of PAD4.	
	Malic acid (**30**)	PAD4	Validated	- Enzymatic inhibition (COLDER assay; BAEE): IC_50_ = 667.10 μM.	/	Weak inhibitor of PAD4.	
	Methyl rosmarinate (**31**)	PAD4	Validated	- Enzymatic inhibition (COLDER assay; BAEE): IC_50_ = 216.40 μM.	/	Moderate inhibitor of PAD4.	
*Bacopa monnieri*	Bacopaside II (**32**)	PAD4	Predicted	- MD: Docking score = 7864; forms H-bonds with Asp350, Glu353, Leu410, Val591, Ala645, Asn648, Val649, and Arg651.	/	Putative direct binder to PAD4 active site.	[118]
*B*. *monnieri*	Bacopaside X (**33**)	PAD4	Predicted	- MD: Docking score = 8432; forms H-bonds with Thr299, Ile354, Val649, and Arg651.	/	Ditto	
Microalgae, bacteria, and fungi	Canthaxanthin (**34**)	PAD4	Predicted	- MD: Docking score = 8544; forms H-bonds with Arg651 and Tyr636.	/	Ditto	
*Broussonetia kazinoki*	Broussonol E (**35**)	PAD4	Predicted	- MD: Docking score = 7962; forms H-bonds with Glu353, His471, Asp473, and Asn648.	/	Ditto	

↑: increase; ↓: decrease.

**Table 4:** Direct inhibitory activities of herbal extracts against the PAD family. All assays were performed using recombinant PAD enzymes.

Natural Source	Natural Product	PAD Isozyme	Assay Type	Substrate	IC_50_/Other Results	Refs.
Moutan Cortex (Tree Peony bark)	Flower ball	PAD4	COLDER assay	BAEE	44.8 μg/mL	[83]
	Flower	PAD4	COLDER assay	BAEE	220.0 μg/mL	
	Leaf	PAD4	COLDER assay	BAEE	145.8 μg/mL	
	Pollen	PAD4	COLDER assay	BAEE	892.6 μg/mL	
	Sead meal	PAD4	COLDER assay	BAEE	30.6 μg/mL	
	Ethyl acetate extract	PAD4	COLDER assay	BAEE	25.13 μg/mL	
	75% ethanol extract	PAD4	COLDER assay	BAEE	53.8 μg/mL	
	Water extract	PAD4	COLDER assay	BAEE	167.0 μg/mL	
Coptidis Rhizoma (Chinese Goldthread rhizome)	Ethyl acetate extract	PAD4	COLDER assay	BAEE	0.72 mg/mL	[113]
			HPLC-UV method	L-Arg	0.47 mg/mL	
	75% Ethanol extract	PAD4	COLDER assay	BAEE	0.23 mg/mL	
			HPLC-UV method	L-Arg	0.20 mg/mL	
	Water extract	PAD4	COLDER assay	BAEE	0.26 mg/mL	
			HPLC-UV method	L-Arg	0.21 mg/mL	
Phellodendri Cortex (Amur Corktree bark)	Ethyl acetate extract	PAD4	COLDER assay	BAEE	2.01 mg/mL	
			HPLC-UV method	L-Arg	0.64 mg/mL	
	75% Ethanol extract	PAD4	COLDER assay	BAEE	1.87 mg/mL	
			HPLC-UV method	L-Arg	0.82 mg/mL	
	Water extract	PAD4	COLDER assay	BAEE	2.04 mg/mL	
			HPLC-UV method	L-Arg	0.90 mg/mL	
*Cratoxylum cochinchinense*	80% Methanol leaf extract	PPAD	Colorimetric assay	BAEE	79% inhibition at 1 mg/mL	[111,113,117]
Salviae Miltiorrhizae Radix Et Rhizoma (Danshen root)	Ethyl acetate extract	PAD4	COLDER assay	BAEE	0.0951 mg/mL	
					0.09 mg/mL	
			HPLC-UV method	L-Arg	0.21 mg/mL	
	75% ethanol extract	PAD4	COLDER assay	BAEE	0.4958 mg/mL	
					0.50 mg/mL	
			HPLC-UV method	L-Arg	0.65 mg/mL	
	Water extract	PAD4	COLDER assay	BAEE	1.496 mg/mL	
					1.41 mg/mL	
			HPLC-UV method	L-Arg	0.80 mg/mL	
Ephedrae Herba (Ephedra stem)	Ethyl acetate extract	PAD4	COLDER assay	BAEE	0.06 mg/mL	[113]
			HPLC-UV method	L-Arg	0.04 mg/mL	
	75% Ethanol extract	PAD4	COLDER assay	BAEE	0.01 mg/mL	
			HPLC-UV method	L-Arg	0.03 mg/mL	
	Water extract	PAD4	COLDER assay	BAEE	0.04 mg/mL	
			HPLC-UV method	L-Arg	0.04 mg/mL	
Cinnamomi Ramulus (Cassia twig)	Ethanol extract	PAD4	Trypsin-assisted chemiluminescent immunoassay	Synthetic peptide	4.4 μg/mL	[87]
			COLDER assay	L-Arg	26.3~119.6 μg/mL (different batches)	
	Ethyl acetate extract	PAD4	COLDER assay	L-Arg	45.3 μg/mL	
	Water extract	PAD4	COLDER assay	L-Arg	118.8 μg/mL	
Cinnamomi Cortex (Cassia bark)	75% Ethanol extract	PAD4	COLDER assay	BAEE	27 μg/mL (vs. MNP@GA@PAD4); 48 μg/mL (vs. free PAD4)	[119]
Forsythiae Fructus (Weeping Forsythia fruit)	Ethyl acetate extract	PAD4	COLDER assay	BAEE	0.9266 mg/mL	[113,117]
					0.93 mg/mL	
			HPLC-UV method	L-Arg	1.32 mg/mL	
	75% ethanol extract	PAD4	COLDER assay	BAEE	0.4743 mg/mL	
					0.47 mg/mL	
			HPLC-UV method	L-Arg	0.99 mg/mL	
	Water extract	PAD4	COLDER assay	BAEE	0.8774 mg/mL	
					0.88 mg/mL	
			HPLC-UV method	L-Arg	0.59 mg/mL	
Sinomenii Caulis (Ovientvine stem)	Ethyl acetate extract	PAD4	COLDER assay	BAEE	0.8774 mg/mL	[113,117]
					0.67 mg/mL	
			HPLC-UV method	L-Arg	0.50 mg/mL	
	75% ethanol extract	PAD4	COLDER assay	BAEE	0.7555 mg/mL	
					0.79 mg/mL	
			HPLC-UV method	L-Arg	0.88 mg/mL	
	Water extract	PAD4	COLDER assay	BAEE	2.033 mg/mL	
					2.03 mg/mL	
			HPLC-UV method	L-Arg	2.20 mg/mL	
Caryophylli Flos (Clove)	75% Ethanol extract	PAD4	COLDER assay	BAEE	48 μg/mL (vs. MNP@GA@PAD4); 32 μg/mL (vs. free PAD4)	[119]
Gardeniae Fructus (Cape Jasmine fruit)	Ethyl acetate extract	PAD4	COLDER assay	BAEE	0.40 mg/mL	[113]
			HPLC-UV method	L-Arg	0.35 mg/mL	
	75% Ethanol extract	PAD4	COLDER assay	BAEE	0.76 mg/mL	
			HPLC-UV method	L-Arg	0.27 mg/mL	
	Water extract	PAD4	COLDER assay	BAEE	2.03 mg/mL	
			HPLC-UV method	L-Arg	0.63 mg/mL	
Scutellariae Radix (Baikal Skullcap root)	Ethyl acetate extract	PAD4	COLDER assay	BAEE	0.42 mg/mL	
			HPLC-UV method	L-Arg	0.48 mg/mL	
	75% Ethanol extract	PAD4	COLDER assay	BAEE	0.61 mg/mL	
			HPLC-UV method	L-Arg	0.76 mg/mL	
	Water extract	PAD4	COLDER assay	BAEE	0.74 mg/mL	
			HPLC-UV method	L-Arg	0.77 mg/mL	

### 3.2. Indirect Modulators of the PAD/Citrullination Axis

In contrast to direct PAD inhibitors, another class of natural products indirectly influences citrullination by regulating PAD expression, PTM, or upstream/downstream signaling pathways. Although these modulators do not directly bind to PADs, they alter PAD activity or the microenvironment via mechanisms such as interfering with inflammatory signaling, modulating immune cell differentiation, or regulating NETosis-related signals. In recent years, a growing body of research has indicated that various natural compounds can indirectly modulate the PAD/citrullination axis, offering unique advantages for treating autoimmune and immune-related disorders.

Curcumin (**36**), a natural polyphenol from turmeric, exhibits therapeutic potential in autoimmune diseases such as RA and SLE [120,121]. It alleviates joint inflammation and reduces citrulline levels in TNF-α-stimulated fibroblast-like synoviocytes from RA patients (FLS-RA), potentially attenuating immune responses [122]. Curcumin also suppresses NF-κB transcriptional activity in FLS cells, reducing inflammatory cytokines and matrix-degrading enzymes. Since PAD2 regulates NF-κB-related proteins [123], curcumin may indirectly affect PAD-mediated citrullination by modulating the inflammatory microenvironment. Additionally, curcumin inhibits macrophage-derived IL-12/IL-18, affecting Th1 differentiation and reducing citrullinated protein generation [124]. In CIA animal models, curcumin alleviates joint swelling and synovial hyperplasia by inhibiting the mTOR signaling pathway and pro-inflammatory cytokines such as IL-1β and TNF-α [125,126], which are upstream signals for NETosis and PAD activation [127,128]. Curcumin also ameliorates lupus nephritis in mouse models by modulating the PI3K/AKT/NF-κB signaling pathway, which regulates neutrophil migration and NETosis [129].

Resveratrol (**37**), a pleiotropic polyphenol found in grapes, cranberries, and peanuts [130], reduces periodontal tissue destruction and local anti-cyclic citrullinated peptide (ACCP) antibody levels in RA patients. Citrullination alters complement activity and induces PGE2, exacerbating periodontal tissue destruction [131]; it may protect by inhibiting this process [132]. It also modulates IL-4 and rheumatoid factor in arthritic rats, alleviating inflammatory symptoms and joint [131]. As IL-4 inhibits pro-inflammatory cytokines such as TNF-α, IL-1β, IL-6, and IL-8 [133], which are upstream signals for PAD activation and NETosis, its upregulation indirectly suppresses the PAD-mediated citrullination process. Resveratrol also acts as a silent information regulator 1 (SIRT1) agonist, inhibiting cathepsin C (CTSC)-induced NET formation by reducing histone H3 citrullination, essential for chromatin decondensation [134].

Epigallocatechin-3-gallate (EGCG, **38**), the main polyphenol in green tea, has anti-inflammatory and antioxidant properties [135]. It inhibits neutrophil elastase (NE), reducing NET formation in PMA-stimulated neutrophils in vitro and in vivo. Since histone H3 citrullination is both NE-dependent and PAD4-mediated, EGCG may indirectly regulate PAD4-dependent citrullination through the inhibition of NE [136]. EGCG also alleviates RA by inhibiting fms-related receptor tyrosine kinase 1 (FLT1) via the PI3K-Akt pathway [137]. FLT1, also known as VEGFR1, a member of the vascular endothelial growth factor receptor family, is upregulated in RA and promotes inflammation [138,139,140,141]; its functional link to PAD4 is evidenced by soluble Flt-1 (sFlt-1)-induced NETosis and pregnancy loss, which is PAD4-dependent [142].

Forsythiasidesare phenylethanoid glycosides found in *Forsythia suspensa*, exhibit a broad range of pharmacological activities [143]. Forsythiaside A (**39**) alleviates UC in mice by inhibiting PAD4 activity and NETosis; it also suppresses PMA-induced PAD4 expression and NETosis in neutrophils [144]. Forsythiaside B (**40**) similarly downregulates PAD4 in peripheral blood neutrophils and ameliorated coagulation dysfunction associated with immune disorders in a sepsis rat model [145]. Since PAD4 is key for histone citrullination during NETosis, forsythiasides A and B are likely to reduce citrullination by downregulating PAD4.

Emodin (**41**), a natural anthraquinone, has anti-inflammatory, antibacterial, anti-allergic, antidiabetic, and immunosuppressive pharmacological effects [146]. It selectively inhibits pro-tumor N2 neutrophils while preserving N1 neutrophil function and reducing NET formation. As NETs depend on PAD4-mediated histone citrullination, emodin may indirectly regulate citrullination by modulating neutrophil phenotypes. Network pharmacology analysis suggests emodin targets TLR, JAK-STAT, and cytokine pathways, which are closely associated with PAD expression and NETosis. Emodin also improves hypercoagulability and inhibits lung carcinogenesis, correlating with reduced N2 neutrophils and NETs [147].

Akebia saponin D (**42**), a saponin from *Dipsacus asper*, has analgesic and anti-inflammatory effects [148]. It improves neurobehavioral outcomes after intracerebral hemorrhage and reduces NET formation by upregulating NTSR1, activating the cAMP signaling pathway, and regulating downstream PKAc activity. This NTSR1/cAMP/PKAc pathway is linked to PAD4-mediated histone citrullination, and PKAc inhibition abolishes the anti-NET effects of akebia saponin D, suggesting indirect regulation of PAD4 activity [149].

**Figure 3:**
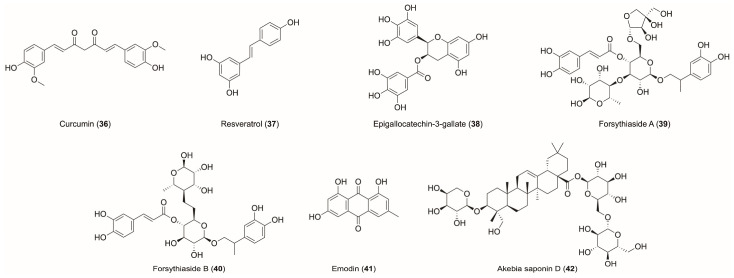
Chemical structures of natural compounds identified as indirect inhibitors of the PAD family.

In summary, although these natural products target different disease models, their core mechanisms revolve around regulating NET formation and the PAD/citrullination axis (Figure 3). As NETs are key in innate immunity and autoimmune diseases such as RA, SLE, and UC, compounds including curcumin, resveratrol, EGCG, forsythiasides, emodin, and akebia saponin D indirectly modulate citrullination by regulating inflammatory signaling, NET formation, or immune cell differentiation. These findings provide new insights for developing immunomodulatory therapies for autoimmune diseases.

### 3.3. Substrate-Directed Intervention

Natural products, owing to their structural complexity and conformational diversity, exhibit unique advantages in targeting shallow protein interfaces. Beyond serving as direct PAD inhibitors or indirect modulators, they also protect key arginine residues on substrates, offering novel therapeutic avenues. While current citrullination interventions (enzyme-centered or indirect strategies) have progressed, they face challenges in clinical translation and precision.

Regarding the enzyme-centered strategy, the five PAD isozymes share high sequence homology, complicating the development of isoform-selective inhibitors [23,150]. PAD enzymes play crucial roles in multiple physiological processes, including gene regulation, embryonic development, and the maintenance of immune homeostasis. Therefore, systemic PAD inhibition may disrupt these functions and lead to off-target side effects [151]. Critically, traditional inhibitors cannot distinguish between pathogenic (e.g., citrullinated fibrin, vimentin, and histone) [152]. As for indirect modulation strategies, though avoiding direct enzyme inhibition, they are hindered by the complexity of upstream signaling networks [153], which complicates precise regulation of specific pathogenic citrullination events.

**Figure 4:**
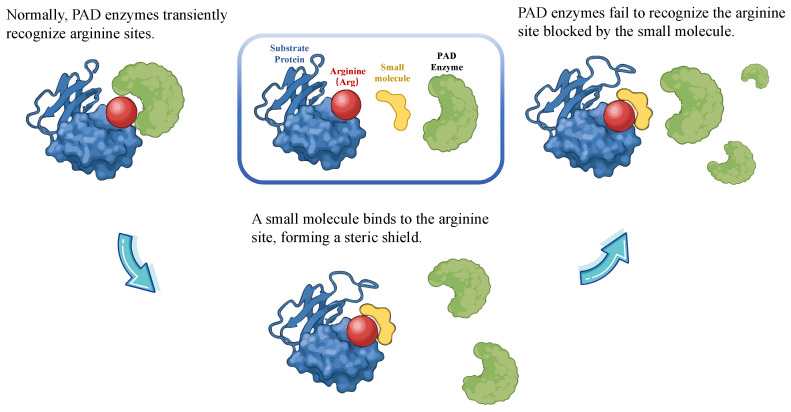
Substrate-directed intervention strategy to suppress PAD-mediated citrullination.

A novel substrate-centered strategy aims to precisely block pathogenic citrullination by protecting key arginine residues, preventing PAD recognition. This aligns with advances in targeting protein–protein interaction (PPI), a feasible strategy by transforming once “undruggable” protein surfaces into therapeutic targets [154]. For instance, venetoclax, the first FDA-approved PPI inhibitor targeting BCL-2 interactions, demonstrates the clinical value of disrupting protein complexes with small molecules [155,156]. Although PAD-substrate recognition is transient, it similarly relies on specific protein surface interfaces [157,158]. Consequently, small molecules can bind critical arginine residues to form a steric shield, blocking PAD access while preserving substrate conformation, and can be regarded as a site-protection mechanism (Figure 4).

Not all arginine residues are equally critical. A glycogen phosphorylase peptide study showed that citrullination at position 16 (but not position 10) reduced catalytic efficiency by approximately 80%, without conformational changes, indicating site-specific recognition [159]. Similarly, protecting core arginine residues on pathogenic antigens (e.g., vimentin) could enable precise anti-citrullination effects.

Natural products serve as ideal candidates for exploring substrate-centered approaches. Their structural complexity and conformational diversity facilitates binding to shallow protein interfaces or grooves. This is exemplified by: kaempferol, which disrupts TNF-α/TNFR1 interaction by occupying the flat protein surface [160,161]; corilagin, a substrate-competitive ERAP1 inhibitor [162]; and macrocyclic compounds with high interface affinity [163]. Notably, we recently found that scopoletin covalently binds vimentin and reduces its citrullination (unpublished), suggesting a protective role for key arginine residues. Although this observation requires further validation, it provides preliminary evidence supporting the feasibility of a substrate-centered strategy.

Despite this preliminary finding, no natural product or small molecules have been experimentally confirmed to inhibit citrullination via direct substrate protection. This represents a clear and significant knowledge gap. Current interventions focus solely on PAD enzymes or indirect regulation of the PAD/citrullination axis, leaving the substrate protection unexplored. Pioneering this frontier could provide alternatives for patients unresponsive to existing therapies and enable precise targeting of pathogenic antigens.

## 4. Challenges and Future Perspectives

### 4.1. Challenges in Direct PAD Inhibition

Direct, enzyme-centric PAD inhibition has a strong rationale but faces translational hurdles. Many advanced PAD inhibitors are covalent electrophiles developed as chemical probes [164]. While effective, they raise concerns about off-target reactivity, idiosyncratic toxicity, and imperfect isoform selectivity in chronic use [165]. A fundamental challenge lies in the risk that broad-spectrum PAD inhibition may interfere with the physiological functions of these enzymes, potentially narrowing the therapeutic window. Furthermore, the efficacy of PAD inhibition is constrained by the biology of citrullination itself, which is tightly regulated by the local microenvironment, including calcium flux, redox state, and NET compartmentalization. Consequently, conventional plasma pharmacokinetics often fail to reflect drug exposure at the disease site. This disconnect arises because physical barriers like NETs and differential concentration gradients between plasma and sites like synovial fluid can limit drug penetration. Achieving effective tissue concentrations under these constraints can narrow the therapeutic window [166,167,168]. Future candidates should therefore be evaluated using a framework that integrates tissue exposure, cellular target engagement, and suppression of citrullination biomarkers [96,168].

### 4.2. Challenges in Indirect Modulation Strategies

In contrast to direct inhibition, the indirect modulation strategy aims to regulate citrullination levels by targeting upstream signaling pathways, immune cell differentiation, or processes like NETosis. While this approach benefits from the multi-pathway regulatory potential of agents like curcumin or resveratrol, it presents a distinct and inherent limitation: the lack of a direct linkage to the specific citrullination event. This indirect nature makes it exceedingly difficult to achieve precise intervention against pathogenic citrullination while sparing its physiological turnover. Consequently, the therapeutic effects are often accompanied by broader, mechanism-based off-target effects rooted in the modulators’ pleiotropic actions. This fundamental issue of precision underscores the need for strategies that can discriminately intercept the citrullination cascade.

### 4.3. The Substrate-Centric Strategy: A Paradigm Shift with New Challenges

The limitations of both direct enzyme inhibition and indirect modulation have catalyzed interest in a substrate-centric strategy, which represents a paradigm shift from inhibiting enzyme activity to disrupting pathogenic protein–protein interactions. This strategy aims to selectively block the formation of the most pathogenic citrullinated neoepitopes by shielding key arginine residues on substrate proteins [5]. However, this approach faces substantial foundational and technical challenges. The first major challenge is the precise identification of disease-specific key arginine modification sites (“hotspots”), which requires deep integration of structural biology, proteomics, and disease pathophysiology. Key steps thereafter include prioritizing driver substrates and citrullination hotspots in disease-relevant contexts, and developing modalities that shield substrates or disrupt enzyme-substrate encounters. This leverages surface-biased discovery platforms, such as fragments, biophysics, and DNA-encoded macrocycles, which are well-suited to target protein–protein interaction interfaces rather than deep enzyme active sites [169]. The subsequent screening for small molecules that can selectively bind to specific arginine regions without perturbing overall protein conformation presents considerable technical difficulty. Furthermore, the choice between covalent and non-covalent binding modes, and their respective impacts on protein function and pharmacokinetics, require systematic investigation. While direct evidence for natural products exhibiting substrate selectivity is still emerging, certain compounds with complex architectures may hold potential for epitope shielding, offering a future avenue for exploration. Proof-of-concept studies, such as engineering LL-37 homoarginine variants, support the feasibility of substrate protection [170,171]. However, assays must rigorously distinguish substrate shielding from direct PAD inhibition and define success as the selective reduction in disease-relevant epitopes with preserved physiological PAD functions.

### 4.4. Cross-Cutting Hurdles and Convergent Future Directions

Across all strategies, translation is hindered by shared challenges in biomarker validation and drug discovery. Current biomarkers, such as CitH3 or MPO-DNA in NETs, are useful but confounded by variations in activation and clearance [167,172,173]. More specific techniques like targeted liquid chromatography–tandem mass spectrometry (LC–MS/MS) for citrullinated peptides require standardization and improved clinical accessibility [174]. In discovery, screening efforts, particularly with redox-active natural products like polyphenols, must rigorously control for artifacts using pan-assay interference compound (PAINS) filters and orthogonal validation such as activity-based protein profiling (ABPP) and cellular thermal shift assays (CETSA) [175,176,177,178]. Looking forward, the field must adopt a holistic perspective: clinically, this may involve combination strategies to balance efficacy and host defense, and biomarker-guided prevention trials in ACPA-positive at-risk populations [179,180]. Scientifically, progress hinges on developing a unified translational framework that seamlessly connects in vitro target engagement, in vivo suppression of pathogenic citrullination signatures, and ultimately, clinical efficacy in defined patient subsets.

## 5. Conclusions

The dysregulated PAD/citrullination axis is a pivotal therapeutic target in immune-mediated diseases. Moving beyond direct enzyme inhibition, this review maps a strategic expansion of the therapeutic landscape, highlighting the unique role of natural products in pioneering these novel avenues. We have systematically catalogued natural products that function as direct inhibitors, indirect modulators, and potential prototypes for a substrate-centric strategy aimed at shielding key arginine residues. This synthesis not only provides a rich repository of chemical leads but also establishes a conceptual framework for multi-layered intervention.

It is imperative to note that the compelling potential outlined here is primarily grounded in preclinical evidence. The translation of these strategies confronts significant, shared challenges: achieving requisite selectivity, validating predictive biomarkers, and ultimately demonstrating efficacy in clinical trials. Therefore, this work serves less as a presentation of immediate solutions and more as a strategic roadmap. The structural and mechanistic diversity of natural products positions them as invaluable tools to address these challenges, guiding the rational development of the next generation of more precise and effective anti-citrullination therapies.

## Figures and Tables

**Table 1:** Characteristics and disease associations of the PAD family.

PAD Isozyme	Primary Expression	Key Physiological Functions	Associated Autoimmune Diseases	Key Pathological Substrates	Refs.
PAD1	Epidermis (differentiated keratinocytes)	Epidermal differentiation; skin barrier formation	Not strongly linked to systemic autoimmunity	Keratin K1/K10; filaggrin	[10,26]
PAD2	Broadly expressed; notably central nervous system (oligodendrocytes/brain), skeletal muscle, immune cells	Protein citrullination in gene regulation and tissue functions; myelin biology	Multiple sclerosis (MS); rheumatoid arthritis (RA) and inflammation	Myelin basic protein; vimentin	[10,26]
PAD3	Hair follicle and epidermis	Hair/skin differentiation; processing of structural proteins	No firm systemic autoimmune association	Trichohyalin; filaggrin	[10,26]
PAD4	Immune cells; especially neutrophils; nuclear localization	NETosis via histone citrullination; chromatin decondensation	RA; systemic lupus erythematosus (SLE) and neutrophil extracellular trap(NET)-driven autoimmunity	Histones (e.g., H3); vimentin; fibrinogen	[10,20,24,26]
PAD6	Oocytes and early embryos	Maternal-effect factor for early development; enzymatic activity unclear vs. PAD1–4	Not established in classic systemic autoimmunity	Substrates not clearly defined	[10,26]

**Table 2:** Pathological roles of PAD isoforms and their synthetic inhibitors in immune diseases.

Disease	Key PAD Isoform	Pathological Substrate	Critical Mechanism	Synthetic PAD Inhibitors	Refs.
RA	PAD2; PAD4	Vimentin; fibrinogen; α-enolase; histones (H2A, H4)	- PAD2/PAD4 upregulation in synovium - Anti-citrullinated protein antibody (ACPA) production against citrullinated antigens - PAD4-driven NET formation fuels inflammation - Cartilage damage via PAD2 activity	Chloramidine (Cl-amidine); BB-Cl-amidine; GSK484; JBI-589; TNF inhibitors; JAK inhibitors	[6,28,33,34,35,36,37,38,39,40,41,42]
MS	PAD2	Myelin basic protein (MBP)	- MBP hypercitrullination (~45% vs. 18% physiological) - Disrupts myelin compaction and charge balance - Triggers Th17 responses via MBP85-99 presentation - PAD2 promoter hypomethylation	Cl-amidine; 2-chloroacetamidine; F-amidine	[43,44,45,46,47,48,49,50,51,52]
SLE	PAD2; PAD4	Histone H3; extracellular proteins	- Neutrophil-derived PAD2/PAD4 release (NET-independent) - Histone citrullination amplifies autoantigen pool - Drives ACPA production and tolerance loss	Cl-amidine; BB-Cl-amidine; GSK484; hydroxychloroquine	[53,54,55,56,57,58,59]
Psoriasis	PAD1; PAD4	Keratin K1; histones	- Reduced citrullination of structural proteins impairs barrier - PAD4 drives NET formation via histone citrullination - Correlates with Th17/neutrophil infiltration	Cl-amidine; BB-Cl-amidine; GSK484; IL-17 inhibitors; IL-23 inhibitors	[5,47,57,60,61,62,63,64,65]
Inflammatory Bowel Disease (IBD)	PAD4 ↑; PAD2 ↓	Mitochondrial creatine kinase 1 (CKMT1); vimentin	- PAD4-mediated CKMT1 citrullination disrupts mitochondrial homeostasis - Citrullinated vimentin as biomarker (lower in UC vs. CD) - Cl-amidine reduces inflammation in models	Cl-amidine; GSK484; anti-TNF agents	[66,67,68,69,70,71]
Type 1 Diabetes (T1D)	PAD2	Glucokinase; glucose-regulated protein 78 (GRP78); glutamate acid decarboxylase 65 (GAD65)	- Selective glucokinase citrullination in pancreas alters enzyme kinetics - PAD2 upregulated in prediabetic islets - PAD inhibition restores insulin secretion impaired by cytokines	YW3-56; Cl-amidine; BB-Cl-amidine	[26,72,73,74,75,76]

**↑**: positive correlation; ↓: negative correlation.

## Data Availability

No new data were created or analyzed in this study.

## Abbreviations

The following abbreviations are used in this manuscript:

RA	Rheumatoid arthritis
MS	Multiple sclerosis
SLE	Systemic lupus erythematosus
PTM	Post-translational modification
PADs	Peptidylarginine deiminases
NETs	Neutrophil extracellular traps
MBP	Myelin basic protein
ACPA	Anti-citrullinated protein antibody
CNS	Central nervous system
IBD	Inflammatory bowel disease
UC	Ulcerative colitis
CD	Crohn’s disease
CKMT1	Mitochondrial creatine kinase 1
Cl-amidine	Chloramidine
T1D	Type 1 diabetes
ICA69	Islet cell autoantigen 69
GAD65	Glutamate acid decarboxylase 65
IA-2	Islet antigen 2
ZnT8	Zinc transporter 8
GRP78	Glucose-regulated protein 78
NOD	Non-obese diabetic
PGG	Pentagalloylglucose
MD	Molecular docking
DARTS	Drug affinity responsive target stability
PQQ	Pyrroloquinoline quinone
EMS	Er Miao San
PHE	Phellodendrine
CitH3	citrullinated histone H3
ATL-I	Atractylenolide-I
CIA	Collagen-induced arthritis
Rb1	Ginsenoside Rb1
FA	Ferulic acid
HMGB1	high mobility group box 1
ICT	Icaritin
FLS-RA	Fibroblast-like synoviocytes from RA patients
ACCP	Anti-cyclic citrullinated peptide
SIRT1	Silent information regulator 1
CTSC	Cathepsin C
EGCG	Epigallocatechin-3-gallate
FLT1	Fms-related receptor tyrosine kinase 1
sFlt-1	Soluble Flt-1
LC–MS/MS	Liquid chromatography–tandem mass spectrometry
PAINS	Pan-assay interference compounds
ABPP	Activity-based protein profiling
CETSA	Cellular thermal shift assay
TCM	Traditional Chinese medicines

## References

[1] Huang M., Ma Z., Luo X., Ren Q. (2025). Epidemiological Burden Assessment of Six Major Immune-Mediated Inflammatory Diseases Based on the Global Burden of Disease Study 2021: Analyses of Age-Standardized Incidence, Prevalence, Mortality, and Disability-Adjusted Life Years. J. Transl. Autoimmun..

[2] Nguyen K.H.H., Le N.V., Nguyen P.H., Nguyen H.H.T., Hoang D.M., Huynh C.D. (2025). Human immune system: Exploring diversity across individuals and populations. Heliyon.

[3] Fugger L., Jensen L.T., Rossjohn J. (2020). Challenges progress, and prospects of developing therapies to treat autoimmune diseases. Cell.

[4] Song Y., Li J., Wu Y. (2024). Evolving understanding of autoimmune mechanisms and new therapeutic strategies of autoimmune disorders. Signal Transduct. Target. Ther..

[5] Ciesielski O., Biesiekierska M., Panthu B., Soszyński M., Pirola L., Balcerczyk A. (2022). Citrullination in the pathology of inflammatory and autoimmune disorders: Recent advances and future perspectives. Cell. Mol. Life Sci..

[6] Witalison E.E., Thompson P.R., Hofseth L.J. (2015). Protein arginine deiminases and associated citrullination: Physiological functions and diseases associated with dysregulation. Curr. Drug Targets.

[7] Rogers G., Simmonds D. (1958). Content of citrulline and other amino-acids in a protein of hair follicles. Nature.

[8] Wesche J., Kühn S., Kessler B.M., Salton M., Wolf A. (2017). Protein arginine methylation: A prominent modification and its demethylation. Cell. Mol. Life Sci..

[9] Zakrzewicz D., Didiasova M., Krueger M., Giaimo B.D., Borggrefe T., Mieth M., Hocke A.C., Zakrzewicz A., Schaefer L., Preissner K.T. (2018). Protein arginine methyltransferase 5 mediates enolase-1 cell surface trafficking in human lung adenocarcinoma cells. Biochim. Biophys. Acta BBA-Mol. Basis Dis..

[10] Zhang X., Xie G., Rao L., Tian C. (2025). Citrullination in health and disease: From physiological function to gene regulation. Genes Dis..

[11] Tilvawala R., Nguyen S.H., Maurais A.J., Nemmara V.V., Nagar M., Salinger A.J., Nagpal S., Weerapana E., Thompson P.R. (2018). The rheumatoid arthritis-associated citrullinome. Cell Chem. Biol..

[12] Kenny E.F., Herzig A., Krüger R., Muth A., Mondal S., Thompson P.R., Brinkmann V., von Bernuth H., Zychlinsky A. (2017). Diverse stimuli engage different neutrophil extracellular trap pathways. eLife.

[13] Guiducci E., Lemberg C., Küng N., Schraner E., Theocharides A.P.A., LeibundGut-Landmann S. (2018). Candida albicans-induced NETosis is independent of peptidylarginine deiminase 4. Front. Immunol..

[14] Singh K., Gupta J.K., Chanchal D.K., Shinde M.G., Kumar S., Jain D., Almarhoon Z.M., Alshahrani A.M., Calina D., Sharifi-Rad J. (2025). Natural products as drug leads: Exploring their potential in drug discovery and development. Naunyn-Schmiedeberg’s Arch. Pharmacol..

[15] Kamel E.M., Allam A.A., Rudayni H.A., Alkhayl F.F.A., Ahmed N.A., Lamsabhi A.M. (2026). Natural Product Modulators of Protein–Protein Interactions: A Comprehensive Review. Phytochem. Anal..

[16] Dzobo K. (2022). The role of natural products as sources of therapeutic agents for innovative drug discovery. Compr. Pharmacol..

[17] Chavanas S., Méchin M.-C., Takahara H., Kawada A., Nachat R., Serre G., Simon M. (2004). Comparative analysis of the mouse and human peptidylarginine deiminase gene clusters reveals highly conserved non-coding segments and a new human gene, PADI6. Gene.

[18] Lange S. (2021). Peptidylarginine deiminases and extracellular vesicles: Prospective drug targets and biomarkers in central nervous system diseases and repair. Neural Regen. Res..

[19] Slade D.J., Fang P., Dreyton C.J., Zhang Y., Fuhrmann J., Rempel D., Bax B.D., Coonrod S.A., Lewis H.D., Guo M. (2015). Protein arginine deiminase 2 binds calcium in an ordered fashion: Implications for inhibitor design. ACS Chem. Biol..

[20] Arita K., Hashimoto H., Shimizu T., Nakashima K., Yamada M., Sato M. (2004). Structural basis for Ca^2+^-induced activation of human PAD4. Nat. Struct. Mol. Biol..

[21] Mondal S., Thompson P.R. (2021). Chemical biology of protein citrullination by the protein A arginine deiminases. Curr. Opin. Chem. Biol..

[22] Dakin L.A., Xing L., Hall J., Ding W., Vajdos F.F., Pelker J.W., Ramsey S., Balbo P., Sahasrabudhe P.V., Banker M.E. (2025). Inhibiting peptidylarginine deiminases (PAD1-4) by targeting a Ca^2+^ dependent allosteric binding site. Nat. Commun..

[23] Kijak-Boćkowska M., Czerwińska J., Owczarczyk-Saczonek A. (2025). Peptidylarginine Deiminases: An Overview of Recent Advances in Citrullination Research. Int. J. Mol. Sci..

[24] Deng Q., Pan B., Alam H.B., Liang Y., Wu Z., Liu B., Mor-Vaknin N., Duan X., Williams A.M., Tian Y. (2020). Citrullinated histone H3 as a therapeutic target for endotoxic shock in mice. Front. Immunol..

[25] Tarcsa E., Marekov L.N., Mei G., Melino G., Lee S.-C., Steinert P.M. (1996). Protein unfolding by peptidylarginine deiminase: Substrate specificity and structural relationships of the natural substrates trichohyalin and filaggrin. J. Biol. Chem..

[26] Yang M.-L., Sodré F.M., Mamula M.J., Overbergh L. (2021). Citrullination and PAD enzyme biology in type 1 diabetes–regulators of inflammation, autoimmunity, and pathology. Front. Immunol..

[27] Costalonga M., Thumbigere-Math V., Herzberg M.C. (2025). Autoimmunity and Periodontitis. J. Periodontal Res..

[28] Darrah E., Andrade F. (2018). Rheumatoid arthritis and citrullination. Curr. Opin. Rheumatol..

[29] Alghamdi M., Alasmari D., Assiri A., Mattar E., Aljaddawi A.A., Alattas S.G., Redwan E.M. (2019). An overview of the intrinsic role of citrullination in autoimmune disorders. J. Immunol. Res..

[30] Nguyen H., James E.A. (2016). Immune recognition of citrullinated epitopes. Immunology.

[31] Van Steendam K., Tilleman K., De Ceuleneer M., De Keyser F., Elewaut D., Deforce D. (2010). Citrullinated vimentin as an important antigen in immune complexes from synovial fluid of rheumatoid arthritis patients with antibodies against citrullinated proteins. Arthritis Res. Ther..

[32] van Venrooij W.J., Pruijn G.J. (2008). An important step towards completing the rheumatoid arthritis cycle. Arthritis Res. Ther..

[33] Cush J.J. (2021). Rheumatoid arthritis: Early diagnosis and treatment. Med. Clin. N. Am..

[34] Damgaard D., Senolt L., Nielsen C.H. (2016). Increased levels of peptidylarginine deiminase 2 in synovial fluid from anti-CCP-positive rheumatoid arthritis patients: Association with disease activity and inflammatory markers. Rheumatology.

[35] Vossenaar E.R., Radstake T.R., van der Heijden A., van Mansum M.A., Dieteren C., de Rooij D.-J., Barrera P., Zendman A.J., van Venrooij W.J. (2004). Expression and activity of citrullinating peptidylarginine deiminase enzymes in monocytes and macrophages. Ann. Rheum. Dis..

[36] Kurowska W., Kuca-Warnawin E.H., Radzikowska A., Maśliński W. (2017). The role of anti-citrullinated protein antibodies (ACPA) in the pathogenesis of rheumatoid arthritis. Cent. Eur. J. Immunol..

[37] Yu H.-C., Lu M.-C. (2019). The roles of anti-citrullinated protein antibodies in the immunopathogenesis of rheumatoid arthritis. Tzu Chi Med. J..

[38] Fan L., He D., Wang Q., Zong M., Zhang H., Yang L., Sun L. (2012). Citrullinated vimentin stimulates proliferation, pro-inflammatory cytokine secretion, and PADI4 and RANKL expression of fibroblast-like synoviocytes in rheumatoid arthritis. Scand. J. Rheumatol..

[39] Song W., Ye J., Pan N., Tan C., Herrmann M. (2021). Neutrophil extracellular traps tied to rheumatoid arthritis: Points to ponder. Front. Immunol..

[40] Chirivi R.G., van Rosmalen J.W., van der Linden M., Euler M., Schmets G., Bogatkevich G., Kambas K., Hahn J., Braster Q., Soehnlein O. (2021). Therapeutic ACPA inhibits NET formation: A potential therapy for neutrophil-mediated inflammatory diseases. Cell. Mol. Immunol..

[41] Clarke J. (2021). Regulatory eosinophils to the rescue. Nat. Rev. Rheumatol..

[42] Riitano G., Spinelli F., Manganelli V., Caissutti D., Capozzi A., Garufi C., Garofalo T., Misasi R., Sorice M., Conti F. (2025). Wnt signaling as a translational target in rheumatoid and psoriatic arthritis. J. Transl. Med..

[43] Ghasemi N., Razavi S., Nikzad E. (2016). Multiple Sclerosis: Pathogenesis, Symptoms, Diagnoses and Cell-Based Therapy. Cell J..

[44] Murúa S.R., Farez M.F., Quintana F.J. (2022). The immune response in multiple sclerosis. Annu. Rev. Pathol. Mech. Dis..

[45] Kamholz J., De Ferra F., Puckett C., Lazzarini R. (1986). Identification of three forms of human myelin basic protein by cDNA cloning. Proc. Natl. Acad. Sci. USA.

[46] Harauz G., Ishiyama N., Hill C.M., Bates I.R., Libich D.S., Farès C. (2004). Myelin basic protein—Diverse conformational states of an intrinsically unstructured protein and its roles in myelin assembly and multiple sclerosis. Micron.

[47] Chirivi R., Van Rosmalen J., Jenniskens G., Pruijn G., Raats J. (2013). Citrullination: A target for disease intervention in multiple sclerosis and other inflammatory diseases?. J. Clin. Cell. Immunol..

[48] Calabrese R., Zampieri M., Mechelli R., Annibali V., Guastafierro T., Ciccarone F., Coarelli G., Umeton R., Salvetti M., Caiafa P. (2012). Methylation-dependent PAD2 upregulation in multiple sclerosis peripheral blood. Mult. Scler. J..

[49] Mastronardi F.G., Noor A., Wood D.D., Paton T., Moscarello M.A. (2007). Peptidyl argininedeiminase 2 CpG island in multiple sclerosis white matter is hypomethylated. J. Neurosci. Res..

[50] Moscarello M.A., Lei H., Mastronardi F.G., Winer S., Tsui H., Li Z., Ackerley C., Zhang L., Raijmakers R., Wood D.D. (2013). Inhibition of peptidyl-arginine deiminases reverses protein-hypercitrullination and disease in mouse models of multiple sclerosis. Dis. Models Mech..

[51] Monreal M.T.M., Hansen B.E., Iversen P.F., Enevold C., Ødum N., Sellebjerg F., Højrup P., von Essen M.R., Nielsen C.H. (2023). Citrullination of myelin basic protein induces a Th17-cell response in healthy individuals and enhances the presentation of MBP85-99 in patients with multiple sclerosis. J. Autoimmun..

[52] Christophorou M.A. (2022). The virtues and vices of protein citrullination. R. Soc. Open Sci..

[53] Siegel C.H., Sammaritano L.R. (2024). Systemic lupus erythematosus: A review. JAMA.

[54] Ziegelasch M., van Delft M.A., Wallin P., Skogh T., Magro-Checa C., Steup-Beekman G.M., Trouw L.A., Kastbom A., Sjöwall C. (2016). Antibodies against carbamylated proteins and cyclic citrullinated peptides in systemic lupus erythematosus: Results from two well-defined European cohorts. Arthritis Res. Ther..

[55] Zhou Y., Chen B., Mittereder N., Chaerkady R., Strain M., An L.-L., Rahman S., Ma W., Low C.P., Chan D. (2017). Spontaneous secretion of the citrullination enzyme PAD2 and cell surface exposure of PAD4 by neutrophils. Front. Immunol..

[56] Sørensen O.E., Borregaard N. (2016). Neutrophil extracellular traps—The dark side of neutrophils. J. Clin. Investig..

[57] Thieblemont N., Wright H.L., Edwards S.W., Witko-Sarsat V. (2016). Human neutrophils in auto-immunity. Semin. Immunol..

[58] Liu Y., Lightfoot Y.L., Seto N., Carmona-Rivera C., Moore E., Goel R., O’Neil L., Mistry P., Hoffmann V., Mondal S. (2018). Peptidylarginine deiminases 2 and 4 modulate innate and adaptive immune responses in TLR-7–dependent lupus. JCI insight.

[59] Hu S.C.-S., Yu H.-S., Yen F.-L., Lin C.-L., Chen G.-S., Lan C.-C.E. (2016). Neutrophil extracellular trap formation is increased in psoriasis and induces human β-defensin-2 production in epidermal keratinocytes. Sci. Rep..

[60] Sieminska I., Pieniawska M., Grzywa T.M. (2024). The immunology of psoriasis—Current concepts in pathogenesis. Clin. Rev. Allergy Immunol..

[61] Clancy K.W., Russell A.-M., Subramanian V., Nguyen H., Qian Y., Campbell R.M., Thompson P.R. (2017). Citrullination/methylation crosstalk on histone H3 regulates ER-target gene transcription. ACS Chem. Biol..

[62] Ishida-Yamamoto A., Takahashi H., Iizuka H., Senshu T., Akiyama K., Nomura K. (2000). Decreased deiminated keratin K1 in psoriatic hyperproliferative epidermis. J. Investig. Dermatol..

[63] Padhi A., Rekha R.S., Benrejdal L., Grundeken M.E., Lourda M., Ehrström M., Eyerich K., Tapia-Páez I., Johansson E.K., Bradley M. (2023). Baricitinib blocks cytokine-mediated downregulation of PAD1 in human keratinocytes: A possible molecular link to the effects of JAK inhibitors in atopic dermatitis. J. Investig. Dermatol..

[64] Méchin M.-C., Takahara H., Simon M. (2020). Deimination and peptidylarginine deiminases in skin physiology and diseases. Int. J. Mol. Sci..

[65] Papadaki G., Kambas K., Choulaki C., Vlachou K., Drakos E., Bertsias G., Ritis K., Boumpas D.T., Thompson P.R., Verginis P. (2016). Neutrophil extracellular traps exacerbate Th1-mediated autoimmune responses in rheumatoid arthritis by promoting DC maturation. Eur. J. Immunol..

[66] Czerwińska J., Owczarczyk-Saczonek A. (2022). The role of the neutrophilic network in the pathogenesis of psoriasis. Int. J. Mol. Sci..

[67] Janssen K.M., Hop H., Vissink A., Dijkstra G., de Smit M.J., Brouwer E., Westra J. (2020). Levels of anti-citrullinated protein antibodies and rheumatoid factor, including iga isotypes, and articular manifestations in ulcerative colitis and crohn’s disease. Int. J. Environ. Res. Public Health.

[68] Wang S., Song Y., Wang Z., Chang X., Wu H., Yan Z., Wu J., He Z., Kang L., Hu W. (2024). Neutrophil-derived PAD4 induces citrullination of CKMT1 exacerbates mucosal inflammation in inflammatory bowel disease. Cell. Mol. Immunol..

[69] Dragoni G., De Hertogh G., Vermeire S. (2021). The role of citrullination in inflammatory bowel disease: A neglected player in triggering inflammation and fibrosis?. Inflamm. Bowel Dis..

[70] Howell K.J., Kraiczy J., Nayak K.M., Gasparetto M., Ross A., Lee C., Mak T.N., Koo B.-K., Kumar N., Lawley T. (2018). DNA methylation and transcription patterns in intestinal epithelial cells from pediatric patients with inflammatory bowel diseases differentiate disease subtypes and associate with outcome. Gastroenterology.

[71] Chumanevich A.A., Causey C.P., Knuckley B.A., Jones J.E., Poudyal D., Chumanevich A.P., Davis T., Matesic L.E., Thompson P.R., Hofseth L.J. (2011). Suppression of colitis in mice by Cl-amidine: A novel peptidylarginine deiminase inhibitor. Am. J. Physiol.-Gastrointest. Liver Physiol..

[72] Mortensen J.H., Godskesen L.E., Jensen M.D., Van Haaften W.T., Klinge L.G., Olinga P., Dijkstra G., Kjeldsen J., Karsdal M.A., Bay-Jensen A.-C. (2015). Fragments of citrullinated and MMP-degraded vimentin and MMP-degraded type III collagen are novel serological biomarkers to differentiate Crohn’s disease from ulcerative colitis. J. Crohns Colitis.

[73] Yang M.-L., Doyle H.A., Clarke S.G., Herold K.C., Mamula M.J. (2018). Oxidative modifications in tissue pathology and autoimmune disease. Antioxid. Redox Signal..

[74] McGinty J.W., Marré M.L., Bajzik V., Piganelli J.D., James E.A. (2015). T cell epitopes and post-translationally modified epitopes in type 1 diabetes. Curr. Diabetes Rep..

[75] Buitinga M., Callebaut A., Sodré F.M.C., Crèvecoeur I., Blahnik-Fagan G., Yang M.-L., Bugliani M., Arribas-Layton D., Marré M., Cook D.P. (2018). Inflammation-induced citrullinated glucose-regulated protein 78 elicits immune responses in human type 1 diabetes. Diabetes.

[76] Crèvecoeur I., Gudmundsdottir V., Vig S., Sodré F.M.C., D’Hertog W., Fierro A.C., Van Lommel L., Gysemans C., Marchal K., Waelkens E. (2017). Early differences in islets from prediabetic NOD mice: Combined microarray and proteomic analysis. Diabetologia.

[77] Yang M.-L., Horstman S., Gee R., Guyer P., Lam T.T., Kanyo J., Perdigoto A.L., Speake C., Greenbaum C.J., Callebaut A. (2022). Citrullination of glucokinase is linked to autoimmune diabetes. Nat. Commun..

[78] Ding M., Bao Y., Liang H., Zhang X., Li B., Yang R., Zeng N. (2024). Potential mechanisms of formononetin against inflammation and oxidative stress: A review. Front. Pharmacol..

[79] Cheng L., Du Z., Yan X., Che M., Zhi G., Ma X., Geng F., Li B. (2025). Formononetin From Sophora flavescens Aiton Alleviates Atopic Dermatitis by Suppressing Neutrophil Extracellular Traps. Phytother. Res..

[80] Wang W., Jiang S., Zhao Y., Zhu G. (2023). Echinacoside: A promising active natural products and pharmacological agents. Pharmacol. Res..

[81] Li Y., Zhu L., Shen Y., Cheng P., Lu K., Qiu W., Zhu M., Zhu C., Wei Z., Lu Y. (2025). Echinacoside inhibits breast cancer metastasis by targeting PAD4 to reduce NETs formation. Res. Sq..

[82] Chang W.-T., Liu P.-Y., Wu S.-N. (2020). High capability of pentagalloylglucose (PGG) in inhibiting multiple types of membrane ionic currents. Int. J. Mol. Sci..

[83] Zhang S., Zhao J., Zhou D., Wei C., Li Y., Zhang Y., Chen X., Dong J., Zhao Z., Wang S. (2025). Discovery of pentagalloylglucose in Moutan Cortex as a highly potent inhibitor of human peptidyl arginine deiminase 4 (PAD4) by UHPLC-MS/MS. J. Pharm. Biomed. Anal..

[84] Ishak N.S.M., Ikemoto K. (2023). Pyrroloquinoline-quinone to reduce fat accumulation and ameliorate obesity progression. Front. Mol. Biosci..

[85] Ma K., Wu Z.-Z., Wang G.-L., Yang X.-P. (2021). Separation and purification of pyrroloquinoline quinone from Gluconobacter oxydans fermentation broth using supramolecular solvent complex extraction. Food Chem..

[86] Shafiq M., Lone Z.R., Bharati P., Mahapatra S., Rai P., Khandelwal N., Gaikwad A.N., Jagavelu K., Hanif K. (2022). Pyrroloquinoline quinone (PQQ) improves pulmonary hypertension by regulating mitochondrial and metabolic functions. Pulm. Pharmacol. Ther..

[87] Zhao J., Zhang S., Dong J., Chen X., Zuo H., Li Y., Gao C., Zhao Z., Qiu X., Tang Z. (2024). Screening and identification of peptidyl arginine deiminase 4 inhibitors from herbal plants extracts and purified natural products by a trypsin assisted sensitive immunoassay based on streptavidin magnetic beads. Talanta.

[88] Och A., Podgórski R., Nowak R. (2020). Biological activity of berberine—A summary update. Toxins.

[89] Wang A.-Q., Yuan Q.-J., Guo N., Yang B., Sun Y. (2021). Research progress on medicinal resources of Coptis and its isoquinoline alkaloids. China J. Chin. Mater. Medica.

[90] Li M., Tian F., Guo J., Li X., Ma L., Jiang M., Zhao J. (2023). Therapeutic potential of Coptis chinensis for arthritis with underlying mechanisms. Front. Pharmacol..

[91] Gu W., Zhang M., Gao F., Niu Y., Sun L., Xia H., Li W., Zhang Y., Guo Z., Du G. (2022). Berberine regulates PADI4-related macrophage function to prevent lung cancer. Int. Immunopharmacol..

[92] Dai X., Yang D., Bao J., Zhang Q., Ding J., Liu M., Ding M., Liu M., Liang J., Jia X. (2020). Er Miao San, a traditional Chinese herbal formula, attenuates complete Freund’s adjuvant-induced arthritis in rats by regulating Th17/Treg cells. Pharm. Biol..

[93] Chen M.-L., Xian Y.-F., Ip S.-P., Tsai S.-H., Yang J.-Y., Che C.-T. (2010). Chemical and biological differentiation of cortex phellodendri chinensis and cortex phellodendri amurensis. Planta Medica.

[94] Tang R., Qin Z.-F., Yin J.-H., Wang J.-Y., Su W.-R., Xuan Z.-H., Chen B., Jia X.-Y. (2025). Er Miao San and its main components phellodendrine and atractylenolide-I exert anti-rheumatoid arthritis effects by inhibiting PAD4 and thereby reducing the formation of NETs. Fitoterapia.

[95] Maugeri N., Campana L., Gavina M., Covino C., De Metrio M., Panciroli C., Maiuri L., Maseri A., D’angelo A., Bianchi M.E. (2014). Activated platelets present high mobility group box 1 to neutrophils, inducing autophagy and promoting the extrusion of neutrophil extracellular traps. J. Thromb. Haemost..

[96] Papayannopoulos V. (2018). Neutrophil extracellular traps in immunity and disease. Nat. Rev. Immunol..

[97] Andersson U., Erlandsson-Harris H. (2004). HMGB1 is a potent trigger of arthritis. J. Intern. Med..

[98] Fan J., He K., Zhang Y., Li R., Yi X., Li S. (2025). HMGB1: New biomarker and therapeutic target of autoimmune and autoinflammatory skin diseases. Front. Immunol..

[99] Zhou Z., Li M., Zhang Z., Song Z., Xu J., Zhang M., Gong M. (2024). Overview of Panax ginseng and its active ingredients protective mechanism on cardiovascular diseases. J. Ethnopharmacol..

[100] Liu J., Lei Z., Wang Z., Wang H., Sun J., Guo D., Luan F., Zou J., Shi Y. (2025). Ethnobotanical usages, phytochemistry, pharmacology, and quality control of chuanxiong rhizoma: A review. J. Ethnopharmacol..

[101] Li J., You Y., Wang Y., Zou J., Xiao S., Yin X., Xu J., Liao F., Zhang H., You Y. (2026). Ferulic acid in combination with ginsenoside Rb1 alleviates myocardial no-reflow by inhibiting platelet HMGB1 release and NET formation. Chin. Med..

[102] Yin J.-Y., Lai M., Yu X.-Y., Su D.-D., Xiong X.-Y., Li Y.-L. (2025). Comprehensive strategies for paclitaxel production: Insights from plant cell culture, endophytic microorganisms, and synthetic biology. Hortic. Res..

[103] Pritzker L., Moscarello M. (1998). A novel microtubule independent effect of paclitaxel: The inhibition of peptidylarginine deiminase from bovine brain. Biochim. Biophys. Acta BBA-Protein Struct. Mol. Enzymol..

[104] Moscarello M., Mak B., Nguyen T., Wood D., Mastronardi F., Ludwin S. (2002). Paclitaxel (Taxol) attenuates clinical disease in a spontaneously demyelinating transgenic mouse and induces remyelination. Mult. Scler. J..

[105] Moscarello M.A., Mastronardi F.G., Wood D.D. (2007). The Role of Citrullinated Proteins Suggests a Novel Mechanism in the Pathogenesis of Multiple Sclerosis. Neurochem. Res..

[106] Bi Z., Zhang W., Yan X. (2022). Anti-inflammatory and immunoregulatory effects of icariin and icaritin. Biomed. Pharmacother..

[107] Mou Z., Chen Y., Hu J., Hu Y., Zou L., Chen X., Liu S., Yin Q., Gong J., Li S. (2024). Icaritin inhibits the progression of urothelial cancer by suppressing PADI2-mediated neutrophil infiltration and neutrophil extracellular trap formation. Acta Pharm. Sin. B.

[108] Nguyen C.N., Trinh B.T., Ngo T.T., Nguyen H.D., Dang S.V., Nguyen L.-H.D. (2022). Two furanoxanthones from the bark of Cratoxylum cochinchinense. Phytochem. Lett..

[109] Montgomery A.B., Kopec J., Shrestha L., Thezenas M.-L., Burgess-Brown N.A., Fischer R., Yue W.W., Venables P.J. (2016). Crystal structure of Porphyromonas gingivalis peptidylarginine deiminase: Implications for autoimmunity in rheumatoid arthritis. Ann. Rheum. Dis..

[110] Tan S.-A., Yam H.C., Cheong S.L., Chow Y.C., Bok C.Y., Ho J.M., Lee P.Y., Gunasekaran B. (2022). Inhibition of Porphyromonas gingivalis peptidyl arginine deiminase, a virulence factor, by antioxidant-rich Cratoxylum cochinchinense: In vitro and in silico evaluation. Saudi J. Biol. Sci..

[111] Panchalingam S., Jayaraman M., Jeyaraman J., Kasivelu G. (2024). Harnessing marine natural products to inhibit PAD4 triple mutant: A structure-based virtual screening approach for rheumatoid arthritis therapy. Arch. Biochem. Biophys..

[112] Yang C.-W., Lee Y.-Z., Hsu H.-Y., Lee S.-J. (2025). Synthesis and evaluation of a novel class of spiro [chromene-2, 2′-indoline] derivatives as potent inhibitors of peptidylarginine deiminase IV to treat rheumatoid arthritis. Eur. J. Med. Chem..

[113] Zhao J., Li Y., Gao C., Zhao Z., Zhang S., Dong J., Zuo H., Chen X., Xie B., Guo Z. (2024). Screening of natural inhibitors against peptidyl arginine deiminase 4 from herbal extracts by a high-performance liquid chromatography ultraviolet-visible based method. J. Chromatogr. A.

[114] Tang W., Ma J., Chen K., Wang K., Chen Z., Chen C., Li X., Wang Y., Shu Y., Zhang W. (2024). Berbamine ameliorates DSS-induced colitis by inhibiting peptidyl-arginine deiminase 4-dependent neutrophil extracellular traps formation. Eur. J. Pharmacol..

[115] Dreyton C.J., Jones J.E., Knuckley B.A., Subramanian V., Anderson E.D., Brown S.J., Fernandez-Vega V., Eberhart C., Spicer T., Zuhl A.M. (2013). Optimization and characterization of a pan protein arginine deiminase (PAD) inhibitor. Probe Reports from the NIH Molecular Libraries Program [Internet].

[116] Ivey A.D., Matthew Fagan B., Murthy P., Lotze M.T., Zeh H.J., Hazlehurst L.A., Geldenhuys W.J., Boone B.A. (2023). Chloroquine reduces neutrophil extracellular trap (NET) formation through inhibition of peptidyl arginine deiminase 4 (PAD4). Clin. Exp. Immunol..

[117] Li Y., Gao C., Zhao J., Zhao Z., Xie B., Zuo H., Zhang S., Dong J., Chen X., Li H. (2024). Screening of peptidyl arginine deiminase 4 inhibitors in traditional herbal medicines. Fitoterapia.

[118] Zhao Z., Wang C., Zhao J., Li Y., Zhang S., Dong J., Zuo H., Ou J., Deng N., Bian Y. (2018). Virtual screening of peptidyl arginine deiminase type 4 inhibiting potential of chosen flavonoids. Res. J. Pharm. Technol..

[119] Zhao Z., Wang C., Zhao J., Li Y., Zhang S., Dong J., Zuo H., Ou J., Deng N., Bian Y. (2024). Immobilized PAD4 enzyme on magnetic nanoparticles for screening natural inhibitors from traditional Chinese medicines. Talanta.

[120] Kou H., Huang L., Jin M., He Q., Zhang R., Ma J. (2023). Effect of curcumin on rheumatoid arthritis: A systematic review and meta-analysis. Front. Immunol..

[121] Chamani S., Moossavi M., Naghizadeh A., Abbasifard M., Majeed M., Johnston T.P., Sahebkar A. (2022). Immunomodulatory effects of curcumin in systemic autoimmune diseases. Phytother. Res..

[122] Ahn J.K., Kim S., Hwang J., Kim J., Lee Y.S., Koh E.-M., Kim K.H., Cha H.-S. (2015). Metabolomic elucidation of the effects of curcumin on fibroblast-like synoviocytes in rheumatoid arthritis. PLoS ONE.

[123] Stachowicz A., Pandey R., Sundararaman N., Venkatraman V., Van Eyk J.E., Fert-Bober J. (2022). Protein arginine deiminase 2 (PAD2) modulates the polarization of THP-1 macrophages to the anti-inflammatory M2 phenotype. J. Inflamm..

[124] Chen Y., Teng Y., Xu P., Wang S. (2024). The role of citrullination modification in CD4+ T cells in the pathogenesis of Immune-Related diseases. Biomolecules.

[125] Deng T., Xu J., Wang Q., Wang X., Jiao Y., Cao X., Geng Q., Zhang M., Zhao L., Xiao C. (2024). Immunomodulatory effects of curcumin on macrophage polarization in rheumatoid arthritis. Front. Pharmacol..

[126] Dai Q., Zhou D., Xu L., Song X. (2018). Curcumin alleviates rheumatoid arthritis-induced inflammation and synovial hyperplasia by targeting mTOR pathway in rats. Drug Des. Dev. Ther..

[127] Sun B., Dwivedi N., Bechtel T.J., Paulsen J.L., Muth A., Bawadekar M., Li G., Thompson P.R., Shelef M.A., Schiffer C.A. (2017). Citrullination of NF-κB p65 promotes its nuclear localization and TLR-induced expression of IL-1β and TNFα. Sci. Immunol..

[128] Wright H.L., Moots R.J., Edwards S.W. (2014). The multifactorial role of neutrophils in rheumatoid arthritis. Nat. Rev. Rheumatol..

[129] Chen X., Gao D., Wang M., Wang L., Hu H., Wen C., Tang Y. (2025). Neutrophil Extracellular Traps in Systemic Lupus Erythematosus: Pathogenic Mechanisms, Crosstalk with Oxidative Stress, and Antioxidant Therapeutic Potential. Antioxidants.

[130] Meng Q., Li J., Wang C., Shan A. (2023). Biological function of resveratrol and its application in animal production: A review. J. Anim. Sci. Biotechnol..

[131] Corrêa M.G., Pires P.R., Ribeiro F.V., Pimentel S.P., Cirano F.R., Napimoga M.H., Casati M.Z., Casarin R.C.V. (2018). Systemic treatment with resveratrol reduces the progression of experimental periodontitis and arthritis in rats. PLoS ONE.

[132] Corrêa M.G., Sacchetti S.B., Ribeiro F.V., Pimentel S.P., Casarin R.C.V., Cirano F.R., Casati M.Z. (2017). Periodontitis increases rheumatic factor serum levels and citrullinated proteins in gingival tissues and alter cytokine balance in arthritic rats. PLoS ONE.

[133] Ogłodek E. (2022). Changes in the serum levels of cytokines: IL-1β, IL-4, IL-8 and IL-10 in depression with and without posttraumatic stress disorder. Brain Sci..

[134] Yu W., Wang Z., Dai P., Sun J., Li J., Han W., Li K. (2023). The activation of SIRT1 by resveratrol reduces breast cancer metastasis to lung through inhibiting neutrophil extracellular traps. J. Drug Target..

[135] Capasso L., De Masi L., Sirignano C., Maresca V., Basile A., Nebbioso A., Rigano D., Bontempo P. (2025). Epigallocatechin gallate (EGCG): Pharmacological properties, biological activities and therapeutic potential. Molecules.

[136] Li H., Qiao C., Zhao L., Jing Q., Xue D., Zhang Y. (2022). Epigallocatechin-3-gallate reduces neutrophil extracellular trap formation and tissue injury in severe acute pancreatitis. J. Leukoc. Biol..

[137] Wang L., Zhao B., Wang J., Zhang D., Ma R., Zhang T., Qi Y., Sheng Y., Hu B., Jin T. (2025). Epigollatecatechin gallate alleviates rheumatoid arthritis through PI3K-Akt pathway by inhibiting FLT1. Int. Immunopharmacol..

[138] Kinghorn K., Gill A., Marvin A., Li R., Quigley K., Singh S., Gore M.T., le Noble F., Gabhann F.M., Bautch V.L. (2024). A defined clathrin-mediated trafficking pathway regulates sFLT1/VEGFR1 secretion from endothelial cells. Angiogenesis.

[139] Meyer A., Zack S.R., Nijim W., Burgos A., Patel V., Zanotti B., Volin M.V., Amin M.A., Lewis M.J., Pitzalis C. (2024). Metabolic reprogramming by Syntenin-1 directs RA FLS and endothelial cell-mediated inflammation and angiogenesis. Cell. Mol. Immunol..

[140] Paradowska-Gorycka A., Sowinska A., Pawlik A., Malinowski D., Stypinska B., Haladyj E., Romanowska-Prochnicka K., Olesinska M. (2017). FLT-1 gene polymorphisms and protein expression profile in rheumatoid arthritis. PLoS ONE.

[141] Yang X., Zhao Y., Wei Q., Zhu X., Wang L., Zhang W., Liu X., Kuai J., Wang F., Wei W. (2024). GRK2 inhibits Flt-1^+^ macrophage infiltration and its proangiogenic properties in rheumatoid arthritis. Acta Pharm. Sin. B.

[142] Erpenbeck L., Chowdhury C.S., Zsengellér Z.K., Gallant M., Burke S.D., Cifuni S., Hahn S., Wagner D.D., Karumanchi S.A. (2016). PAD4 deficiency decreases inflammation and susceptibility to pregnancy loss in a mouse model. Biol. Reprod..

[143] Yang H.-X., Liu Q.-P., Zhou Y.-X., Chen Y.-Y., An P., Xing Y.-Z., Zhang L., Jia M., Zhang H. (2022). Forsythiasides: A review of the pharmacological effects. Front. Cardiovasc. Med..

[144] Wang Z., Yan W., Lin X., Qin G., Li K., Jiang L., Li X., Xiao X., Luo T., Hou Y. (2025). Forsythiaside A alleviates ulcerative colitis and inhibits neutrophil extracellular traps formation in the mice. Phytother. Res..

[145] He W., Xi Q., Cui H., Zhang P., Huang R., Wang T., Wang D. (2022). Forsythiaside B ameliorates coagulopathies in a rat model of sepsis through inhibition of the formation of PAD4-dependent neutrophil extracellular traps. Front. Pharmacol..

[146] Cheng L., Chen J., Rong X. (2022). Mechanism of Emodin in the Treatment of Rheumatoid Arthritis. Evid.-Based Complement. Altern. Med..

[147] Li Z., Lin Y., Zhang S., Zhou L., Yan G., Wang Y., Zhang M., Wang M., Lin H., Tong Q. (2019). Emodin regulates neutrophil phenotypes to prevent hypercoagulation and lung carcinogenesis. J. Transl. Med..

[148] Gong L.-L., Yang S., Liu H., Zhang W., Ren L.-L., Han F.-F., Lv Y.-L., Wan Z.-R., Liu L.-H. (2019). Anti-nociceptive and anti-inflammatory potentials of Akebia saponin D. Eur. J. Pharmacol..

[149] Gu L., Ye L., Chen Y., Deng C., Zhang X., Chang J., Feng M., Wei J., Bao X., Wang R. (2024). Integrating network pharmacology and transcriptomic omics reveals that akebia saponin D attenuates neutrophil extracellular traps-induced neuroinflammation via NTSR1/PKAc/PAD4 pathway after intracerebral hemorrhage. FASEB J..

[150] Barasa L., Thompson P.R. (2023). Protein citrullination: Inhibition, identification and insertion. Philos. Trans. R. Soc. B Biol. Sci..

[151] Bashir F., Awais H., Waseem A., Shahzad A., Babar A., Ali S.A., Shafiq L., Ahmed M. (2025). Structural and mechanistic insights into peptidylarginine deiminase (PAD2/PAD4) mediated citrullination and therapeutic targeting: A review. Int. J. Biol. Macromol..

[152] Mansouri P., Mansouri P., Behmard E., Najafipour S., Kouhpayeh S.A., Farjadfar A. (2024). Peptidylarginine deiminase (PAD): A promising target for chronic diseases treatment. Int. J. Biol. Macromol..

[153] Zehra N., Uddin Z., Zada M.I.A., Ikram M. (2025). Inflammatory signaling and immune response. Cell Signaling.

[154] Cierpicki T., Grembecka J. (2025). Targeting Protein–Protein Interactions in Hematologic Malignancies. Annu. Rev. Pathol. Mech. Dis..

[155] Shin W.-H., Kumazawa K., Imai K., Hirokawa T., Kihara D. (2020). Current challenges and opportunities in designing protein–protein interaction targeted drugs. Adv. Appl. Bioinform. Chem..

[156] Lu H., Zhou Q., He J., Jiang Z., Peng C., Tong R., Shi J. (2020). Recent advances in the development of protein–protein interactions modulators: Mechanisms and clinical trials. Signal Transduct. Target. Ther..

[157] Perkins J.R., Diboun I., Dessailly B.H., Lees J.G., Orengo C. (2010). Transient protein-protein interactions: Structural, functional, and network properties. Structure.

[158] Ghadie M.A., Xia Y. (2022). Are transient protein-protein interactions more dispensable?. PLoS Comput. Biol..

[159] Bartleson C., Luo S., Graves D.J., Martin B.L. (2000). Arginine to citrulline replacement in substrates of phosphorylase kinase. Biochim. Biophys. Acta BBA-Protein Struct. Mol. Enzymol..

[160] Wang S., Shi X., Li J., Huang Q., Ji Q., Yao Y., Wang T., Liu L., Ye M., Deng Y. (2022). A Small Molecule Selected from a DNA-Encoded Library of Natural Products That Binds to TNF-α and Attenuates Inflammation In Vivo. Adv. Sci..

[161] Zhang G., Zhang J., Gao Y., Li Y., Li Y. (2022). Strategies for targeting undruggable targets. Expert Opin. Drug Discov..

[162] Sun X., Zhou Y., Yu S., Liu X., Wu J., Zhou Y., Bai J., Li D., Xu H., Cao H. (2025). Corilagin regulates antigen processing and presentation by directly binding to inhibit ERAP1. Int. Immunopharmacol..

[163] Schäfer S.C., Voll A.M., Bracher A., Ley S.V., Hausch F. (2024). Antascomicin B stabilizes FKBP51-Akt1 complexes as a molecular glue. Bioorganic Med. Chem. Lett..

[164] Nemmara V.V., Thompson P.R. (2018). Development of activity-based proteomic probes for protein citrullination. Act.-Based Protein Profiling.

[165] Causey C.P., Jones J.E., Slack J.L., Kamei D., Jones L.E., Subramanian V., Knuckley B., Ebrahimi P., Chumanevich A.A., Luo Y. (2011). The development of N-α-(2-carboxyl) benzoyl-N^5^-(2-fluoro-1-iminoethyl)-l-ornithine amide (o-F-amidine) and N-α-(2-carboxyl) benzoyl-N^5^-(2-chloro-1-iminoethyl)-l-ornithine amide (o-Cl-amidine) as second generation protein arginine deiminase (PAD) inhibitors. J. Med. Chem..

[166] Vossenaar E.R., Zendman A.J., van Venrooij W.J., Pruijn G.J. (2003). PAD, a growing family of citrullinating enzymes: Genes, features and involvement in disease. BioEssays.

[167] Brinkmann V., Reichard U., Goosmann C., Fauler B., Uhlemann Y., Weiss D.S., Weinrauch Y., Zychlinsky A. (2004). Neutrophil extracellular traps kill bacteria. Science.

[168] Lewis H.D., Liddle J., Coote J.E., Atkinson S.J., Barker M.D., Bax B.D., Bicker K.L., Bingham R.P., Campbell M., Chen Y.H. (2015). Inhibition of PAD4 activity is sufficient to disrupt mouse and human NET formation. Nat. Chem. Biol..

[169] Franzini R.M., Randolph C. (2016). Chemical space of DNA-encoded libraries: Miniperspective. J. Med. Chem..

[170] Bryzek D., Golda A., Budziaszek J., Kowalczyk D., Wong A., Bielecka E., Shakamuri P., Svoboda P., Pohl J., Potempa J. (2020). Citrullination-resistant LL-37 is a potent antimicrobial agent in the inflammatory environment high in arginine deiminase activity. Int. J. Mol. Sci..

[171] Wong A., Bryzek D., Dobosz E., Scavenius C., Svoboda P., Rapala-Kozik M., Lesner A., Frydrych I., Enghild J., Mydel P. (2018). A novel biological role for peptidyl-arginine deiminases: Citrullination of cathelicidin LL-37 controls the immunostimulatory potential of cell-free DNA. J. Immunol..

[172] Gertsch J. (2011). Botanical drugs, synergy, and network pharmacology: Forth and back to intelligent mixtures. Planta Medica.

[173] Yang Y., Guo L., Yu J., Peng W., Wang Q., Wei L., Chen X., Min C., Jiang X., Zhu S. (2025). Da-Yuan-Yin decoction suppresses NETs formation, inhibits the IL-23/JAK2/STAT3 signalling pathway, and modulates metabolic profiles and gut microbiota composition in damp-heat syndrome. Ann. Med..

[174] Leitner A. (2016). Cross-linking and other structural proteomics techniques: How chemistry is enabling mass spectrometry applications in structural biology. Chem. Sci..

[175] Baell J.B., Holloway G.A. (2010). New substructure filters for removal of pan assay interference compounds (PAINS) from screening libraries and for their exclusion in bioassays. J. Med. Chem..

[176] Dahlin J.L., Nissink J.W.M., Strasser J.M., Francis S., Higgins L., Zhou H., Zhang Z., Walters M.A. (2015). PAINS in the assay: Chemical mechanisms of assay interference and promiscuous enzymatic inhibition observed during a sulfhydryl-scavenging HTS. J. Med. Chem..

[177] Aldrich C., Bertozzi C., Georg G.I., Kiessling L., Lindsley C., Liotta D., Merz K.M., Schepartz A., Wang S. (2017). The Ecstasy and Agony of Assay Interference Compounds.

[178] Cravatt B.F., Wright A.T., Kozarich J.W. (2008). Activity-based protein profiling: From enzyme chemistry to proteomic chemistry. Annu. Rev. Biochem..

[179] Fraenkel L., Bathon J.M., England B.R., Clair E.W.S., Arayssi T., Carandang K., Deane K.D., Genovese M., Huston K.K., Kerr G. (2021). American College of Rheumatology guideline for the treatment of rheumatoid arthritis. Arthritis Rheumatol..

[180] Tanaka Y., Luo Y., O’Shea J.J., Nakayamada S. (2022). Janus kinase-targeting therapies in rheumatology: A mechanisms-based approach. Nat. Rev. Rheumatol..

